# Luminescent Ln^3+^-based silsesquioxanes with a β-diketonate antenna ligand: toward the design of efficient temperature sensors

**DOI:** 10.3389/fchem.2024.1379587

**Published:** 2024-04-03

**Authors:** Gautier Félix, Alena N. Kulakova, Saad Sene, Victor N. Khrustalev, Miguel A. Hernández-Rodríguez, Elena S. Shubina, Tristan Pelluau, Luís D. Carlos, Yannick Guari, Albano N. Carneiro Neto, Alexey N. Bilyachenko, Joulia Larionova

**Affiliations:** ^1^ CNRS, ENSCM, University Montpellier, Montpellier, France; ^2^ Nesmeyanov Institute of Organoelement Compounds, Russian Academy of Sciences, Moscow, Russia; ^3^ Peoples’ Friendship University of Russia (RUDN University), Moscow, Russia; ^4^ Zelinsky Institute of Organic Chemistry, Russian Academy of Sciences, Moscow, Russia; ^5^ Phantom-g, Physics Department and CICECO—Aveiro Institute of Materials, University of Aveiro, Aveiro, Portugal; ^6^ Departamento de Física, Universidad de La Laguna San Cristóbal de La Laguna, Santa Cruz de Tenerife, Spain

**Keywords:** thermometry, silsesquioxanes, lanthanides, luminescence, magnetism, energy transfer

## Abstract

We report the synthesis and single-crystal X-ray diffraction, magnetic, and luminescence measurements of a novel family of luminescent cage-like tetranuclear silsesquioxanes (PhSiO_1.5_)_8_(LnO_1.5_)_4_(O)(C_5_H_8_O_2_)_6_(EtOH)_2_(CH_3_CN)_2_⋅2CH_3_CN (where Ln = Tb, **1**; Tb/Eu, **2**; and Gd, **3**), featuring seven-coordinated lanthanide ions arranged in a one-capped trigonal prism geometry. Compounds **1** and **2** exhibit characteristic Tb^3+^ and Tb^3+^/Eu^3+^-related emissions, respectively, sensitized by the chelating antenna acetylacetonate (acac) ligands upon excitation in the UV and visible spectral regions. Compound **3** is used to assess the energies of the triplet states of the *acac* ligand. For compound **1**, theoretical calculations on the intramolecular energy transfer and multiphonon rates indicate a thermal balance between the ^5^D_4_ Stark components, while the mixed Tb^3+^/Eu^3+^ analog **2**, with a Tb:Eu ratio of 3:1, showcases intra-cluster Tb^3+^-to-Eu^3+^ energy transfer, calculated theoretically as a function of temperature. By utilizing the intensity ratio between the ^5^D_4_→^7^F_5_ (Tb^3+^) and ^5^D_0_→^7^F_2_ (Eu^3+^) transitions in the range 11–373 K, we demonstrate the realization of a ratiometric luminescent thermometer with compound **2**, operating in the range 11–373 K with a maximum relative sensitivity of 2.0% K^−1^ at 373 K. These findings highlight the potential of cage-like silsesquioxanes as versatile materials for optical sensing-enabled applications.

## 1 Introduction

Luminescent coordination complexes containing trivalent lanthanide ions (Ln^3+^) have attracted significant attention over several decades due to tremendous perspectives in different applications, including bioimaging and biosensing (Eliseeva and Bünzli, 2009; Ning et al., 2019), light-emitting technology (Bünzli, 2019; Costa et al., 2024), smart windows (Choi et al., 2017; Fernandes et al., 2018; Costa et al., 2024), nano-thermometry (Allison, 2019; Brites et al., 2019; Brites et al., 2023), detection of molecules and ions (Eliseeva and Bünzli, 2009; Allison, 2019; Brites et al., 2023), cell labeling (Aulsebrook et al., 2018; Bodman and Butler, 2021). These compounds exhibit distinctive photophysical characteristics, manifesting as prolonged emission with lifetimes extending up to milliseconds. This extended emission attributed to the 4f–4f transitions occurs in the visible and/or near-infrared (NIR) spectral domains depending on the nature of the Ln^3+^ ion. Moreover, it usually includes narrow emission lines, significant ligand-induced Stokes shifts, and high quantum yields. For these reasons, the coordination chemistry of Ln^3+^ ions employed in association with various ligands to design mono- and polynuclear compounds of various topologies with optimized optical properties has been the subject of huge development during recent decades (Bünzli, 2010; Bünzli, 2015; SeethaLekshmi et al., 2017; Liu et al., 2020; Monteiro, 2020; Bernot et al., 2021). In particular, extensive effort has been focused on the synthesis of highly luminescent, photo- and thermostable complexes valuable for the above-mentioned applications (Kaczmarek et al., 2019).

However, directly exciting the 4f levels poses a challenge as the majority of the f-f transitions are prohibited by the Laporte rule, resulting in low molar absorption coefficients. This constraint can be surmounted by the tune of organic chromophores as ligands to generate the well-known “antenna effect,” yielding highly emissive complexes. In these systems, chromophore ligands, typically excited in the UV or, less frequently, in the visible spectral domains, allow the energy transfer from the ligand triplet excited state to the emitting level of the Ln^3+^ ion. Consequently, this process induces a radiative decay in the visible or NIR spectral regions depending on the Ln^3+^ ion used (Bünzli, 2015). Note that an alternative mechanism has been documented, which entails the energy transfer from the first excited singlet state of the ligand to the 4f levels (Alaoui, 1995; Yang et al., 2004; Kasprzycka et al., 2017; Gregório et al., 2019; Moura Jr. et al., 2021a; Manzur et al., 2023). Hence, achieving efficient energy transfer requires a meticulous alignment of energy levels and adherence to selection rules among various excited states. This optimization can be accomplished through a careful selection of ligands and Ln^3+^ ions, coupled with the strategic design of the metal center geometry within a suitable ligand environment (Carneiro Neto et al., 2019). Furthermore, note that improved luminescence has been documented in complexes featuring an asymmetric Ln^3+^ coordination environment, notably in a seven-coordinated geometry (Miyata et al., 2009; Miyata et al., 2012; Yanagisawa et al., 2015; Ferreira da Rosa et al., 2020; Aquino LE do et al., 2021).

A significant breakthrough in Ln^3+^-based luminescent coordination compounds lies in their application as temperature sensors, enabling remote temperature measurements through emission monitoring. This approach offers temperature sensing with high spatial precision (below 10 µm), temporal (time scale) and thermal resolutions (0.1 K) within short acquisition times (less than 10 µs), and high relative thermal sensitivity (S_r_ > 1% K^−1^) (Brites et al., 2012; Jaque and Vetrone, 2012; Brites et al., 2016; Brites et al., 2019; Brites et al., 2023). It shows enormous potential in a wide range of applications ranging from biology and medicine to cryogenics (Cui et al., 2012; Bettinelli et al., 2015; Dramićanin, 2020). Starting from the first example showing the possibility of the real-time observation of thermogenesis in a single HeLa cell using the simple mononuclear Eu(tta)_3_ complex (where tta^−^ is 3-thenoyltrifluoroacetonate) (Suzuki et al., 2007), numerous Ln^3+^-based compounds have been investigated for this purpose. A particular emphasis in this field has been placed on the design of ratiometric thermometers, in which a luminescence intensity ratio (LIR) of two constituent Ln^3+^ ions is used as a signal permitting a self-referencing emissive system (Brites et al., 2010). The most used complexes contain a Tb^3+^/Eu^3+^ pair due to the high quantum yield of these ions, for which different parameters can be optimized to increase the performance of the luminescent thermometers (Cui et al., 2012; Rocha et al., 2016; Lyubov et al., 2022). In particular, molecular cluster-aggregates have shown high potential for this purpose, attributed to a rigid metal core of high nuclearity, precise coordination environment, and tunable energy transfer. This is achieved through the precise control of the ratio of the ions and the distance between them, as well as their structural organization, ion environment, and the nature of ligands (Calado et al., 2023; Gálico et al., 2023). A particular family of these molecular cluster-aggregates, luminescent Ln^3+^-based silsesquioxanes, provides a compelling alternative to coordination complexes, offering high photothermal and chemical stabilities, particularly pertinent with increasing temperature for thermometry applications.

Cage-like Ln^3+^-based metallasilsesquioxanes are an exciting family of compounds presenting the combination of Ln^3+^ ions with the silsesquioxane repeating subunits, (RSiO_1.5_)_n_ (where *n* = 6, 8, 10, 12, … ). The latter permits the construction of inorganic Si-O-Si skeletons as a basic structural unit, realizing cyclic and polycyclic types of matrixes, which offer these architectures the chemical stability, mechanical robustness, thermal stability, and possibility to form cage-like topology. Moreover, they possess oxygen atoms able to coordinate Ln^3+^ ions and, therefore, integrate and specially organize them inside the cage-like rigid framework. Moreover, terminal ligands may also be coordinated with the metal ions to complete their coordination environment, bringing additional functionalities. These architectures have mainly been investigated as molecule-based models for catalysis (Herrmann et al., 1994; Shchegolikhina et al., 1996; Annand et al., 1999; Annand and Aspinall H, 2000; Arnold et al., 2001; Lorenz et al., 2002; Lorenz et al., 2004; Wu et al., 2009; Marchesi et al., 2014; Davies et al., 2017; Sheng et al., 2022a; Sheng et al., 2022b), but recently, their magnetic (Sheng et al., 2022b) and optical properties (Sheng et al., 2022a) have also been investigated. Recently, we reported the first examples of luminescent Tb^3+^, Eu^3+^, and Dy^3+^-based metallasilsesquioxanes presenting unusual (for metallasilsesquioxanes) anionic prism-like structures. These structures encompass four Ln^3+^ ions linked through oxygen atoms and situated between two cyclic tetraphenylcyclotetrasiloxanolate moieties (Kulakova et al., 2020; Kulakova et al., 2021; Nigoghossian et al., 2021; Félix et al., 2023a; Félix et al., 2023b). They present Ln^3+^ characteristic luminescence and interesting magnetic properties, depending on the nature of the ion, chemical robustness, and thermal stability. Moreover, we demonstrated that anionic cages containing mixed Tb^3+^/Eu^3+^ exhibited a tunable thermosensitive Tb^3+^-to-Eu^3+^ energy transfer and proposed them as an efficient temperature sensor operating in the range 300–373 K with good linearity and repeatability. Remarkably, these compounds presented an important stability to photobleaching at a relatively high working temperature (100°C) due to the presence of the siloxane matrix, which played a protective role. However, the Ln^3+^ ions in these structures are not coordinated with antenna ligands, and the excitation was performed directly in the 4f levels, which is not optimal to achieve highly luminescent materials.

A major objective of the present work consists of the sensitization of Ln^3+^ luminescence in the silsesquioxane cages through the coordination of an antenna ligand. The most popular chelating acetylacetonate (*acac*) antenna has been used as a terminal ligand for this purpose (de Sá et al., 2000; Arnold et al., 2001; Eliseeva and Bünzli, 2009; Bünzli and Eliseeva, 2013; Gálico et al., 2023). Here, we report the synthesis, crystal structures, magnetic properties, and luminescence investigations of three new tetranuclear complexes (PhSiO_1.5_)_8_(LnO_1.5_)_4_(O)(C_5_H_8_O_2_)_6_(EtOH)_2_(CH_3_CN)_2_⋅2CH_3_CN (where Ln = Tb (**1**), Tb/Eu (**2**), and Gd (**3**)) by introducing a terminal chelating ligand *acac* coordinated with the Ln^3+^ ions. Notably, this not only permits to afford an antenna effect and sensitizes the characteristic 4f luminescence but also induces an important change in the geometry of the ions from distorted antiprism (octa-coordinated) to a pentagonal bipyramidal geometry (seven-coordinated), which can positively impact their photophysical properties (Ferreira da Rosa et al., 2020). Complexes **1** and **2** present high Tb^3+^ or Tb^3+^/Eu^3+^ characteristic emissions and paramagnetic properties, while complex **3** has been used to assess the energies of the triplet states of the *acac* ligand. Theoretical calculations on the intramolecular energy transfer and multiphonon rates for the Tb^3+^-based compound indicate a thermal balance between Stark components of the ^5^D_4_ level. The mixed Tb^3+^/Eu^3+^ analog showcases intra-cluster Tb^3+^-to-Eu^3+^ energy transfer and may be used for efficient temperature sensing with good stability, sensibility (S_r_ = 2.0 % K^−1^ at 373 K), and repeatability after several heating/cooling cycles.

## 2 Materials and methods

Phenyltrimethoxysilane (98%), Et_4_NCl (≥98%), Eu(NO_3_)_3_⋅6H_2_O (99.9% trace metal basis), Tb(NO_3_)_3_⋅6H_2_O (99.9% trace metal basis), ethanol, acetylacetonate, and acetonitrile were purchased from Merck and used as received.

### 2.1 Synthesis

Compounds **1**–**3** were synthesized in a similar way. A mixture of PhSi(OMe)_3_ and NaOH was dissolved in 30 mL of ethanol. The resulting solution was heated to reflux for 1.0 h. Afterward, Tb(NO_3_)_3_⋅6H_2_O for compound **1** (or the mixture of Tb(NO_3_)_3_⋅6H_2_O and Eu(NO_3_)_3_⋅6H_2_O, in a 3:1 ratio, for compound **2**, or Gd(NO_3_)_3_⋅5H_2_O for compound **3**) and sodium acetylacetonate dissolved in 30 mL of CH_3_CN were added at once. The resulting mixture was heated to reflux for 3.0 h. Filtration of the mixture from the insoluble part provided a non-colored solution. Slow evaporation of solvents (ethanol/CH_3_CN) provided, in 5–10 days, many crystalline materials. The single crystals suitable for single-crystal X-ray diffraction were collected. The crystal products were dried in a vacuum to perform elemental analysis and calculate the yield.

(PhSiO_1.5_)_8_(TbO_1.5_)_4_(O)(C_5_H_8_O_2_)_6_(EtOH)_2_(CH_3_CN)_2_⋅2CH_3_CN 1. Reactant loadings: PhSi(OMe)_3_ (0.186 g, 0.8 mmol), NaOH (0.032 g, 0.8 mmol), Tb(NO_3_)_3_∙6H_2_O (0.174 g, 0.4 mmol), and sodium acetylacetonate (0.049 g, 0.4 mmol). Yield = 30% (0.068 g).

Anal. calcd for C_78_H_88_Tb_4_O_30_Si_8_: % C 39.60, % H 3.75. Found: % C 39.54, % H 3.71. IR in KBr pellets (cm^−1^): 3443 (w), 3071 (s), 3048 (s), 1592 (s), 1519 (s), 1384 (w), 1129 (s), 1051 (w), 1027 (w), 952 (s), 945 (w), 835 (s), 745 (s), 700 (s), 676 (s), 576 (s), 547 (s), and 494 (s). (PhSiO_1.5_)_8_(TbO_1.5_)_3_(EuO_1.5_)(O)(C_5_H_8_O_2_)_6_(EtOH)_2_(CH_3_CN)_2_⋅2CH_3_CN 2. Reactant loadings: PhSi(OMe)_3_ (0.186 g, 0.8 mmol), NaOH (0.032 g, 0.8 mmol), Tb(NO_3_)_3_∙6H_2_O (0.131 g, 0.3 mmol), Eu(NO_3_)_3_∙5H_2_O (0.043 g, 0.1 mmol), and sodium acetylacetonate (0.049 g, 0.4 mmol). Yield = 25% (0.057 g).

Anal. calcd for C_78_H_88_Eu_3_O_30_Si_8_Tb: % C 39.95, % H 3.78. Found: % C 39.89, % H 3.73. EDS analysis: Tb/Eu ratio 22.1/7.4. IR in KBr pellets (cm^−1^): 3619 (s), 3365 (w), 3071 (s), 3048 (s), 1592 (s), 1518 (s), 1429 (s), 1384 (w), 1266 (s), 1129 (s), 1052 (m), 950 (s), 745 (s), 701 (s), 676 (s), 576 (s), and 494 (s).

(PhSiO_1.5_)_8_(GdO_1.5_)_4_(O)(C_5_H_8_O_2_)_6_(EtOH)_2_(CH_3_CN)_2_⋅2CH_3_CN 3. Reactant loadings: PhSi(OMe)_3_ (0.186 g, 0.8 mmol), NaOH (0.032 g, 0.8 mmol), Gd(NO_3_)_3_•5H_2_O (0.173 g, 0.4 mmol), and sodium acetylacetonate (0.049 g, 0.4 mmol). Yield = 39% (0.092 g).

Anal. calcd for C_78_H_88_Gd_**4**_O_30_Si_8_: % C 39.71, % H 3.76. Found: % C 39.63, % H 3.72. IR in KBr pellets (cm^−1^): 3439 (w), 3066 (s), 3045 (s), 1596 (s), 1517 (s), 1390 (w), 1121 (s), 1060 (w), 1025 (w), 950 (s), 940 (w), 833 (s), 741 (s), 690 (s), 674 (s), 579 (s), 544 (s), and 491 (s).

### 2.2 Characterization

IR spectra (KBr pellets) were recorded using a PerkinElmer Spectrum Two FT-IR spectrometer. The quantification of Eu, Tb, and Si was performed using a scanning electron microscope and energy-dispersive X-ray analysis (SEM-EDX) on a FEI Quanta FEG 200 instrument. The powders were deposited on an adhesive carbon film and analyzed under vacuum. The heavy elements were quantified using INCA software, with a dwell time of 3 µs.

The emission and excitation spectra were at first evaluated at room (298 K) and low (77 K) temperatures using an Edinburgh FLS-920 spectrofluorimeter. The excitation source was a 450-W Xe arc lamp. The spectra were corrected for the detection and optical spectral response of the spectrofluorimeter. In the second step, the emission spectra were measured as a function of the temperature. The temperature setup included a thermal element (Heidolph, MR Hei-Tec [EU], 825 W, plate diameter 145 mm), a thermal camera (Optris PI 450i, accuracy ± 0.01°C), an excitation source, and a detector. The powder sample was placed on a cover glass (14 mm diameter) at the center of the heating source. The thermal camera was positioned at an angle of 30° relative to the sample to work as a temperature standard controller. A UV LED operating at 365 nm (Thorlabs M365L2) was used to excite the samples (I = 0.7A) by irradiating at 15 mm from the sample surface.

The spectrometric detector and the excitation source were coupled using a multimode fiber. The fiber excitation output and detector were located at the top of the sample. A long-pass filter (in-line fiber optic filter mount, Thorlabs, FOFMS/M, 450 nm, 20 µm) was placed in the light path between the sample and detector to avoid artifacts arising from the excitation source. The emission spectra were recorded in the temperature range from 300 to 376 K. At each temperature step, 10 min was provided to allow the temperature to stabilize, and then, 10 emission spectra were recorded from an average of 10 consecutive spectra with an integration time of 100 ms.

The UV–visible–NIR absorption spectrum was measured using a Specord 210 Plus spectrophotometer (Analytik Jena AG, Germany). Magnetic susceptibility data were collected using a Quantum Design MPMS-XL SQUID magnetometer working between 1.8 and 350 K with a magnetic field up to 7 T. The sample was prepared under an ambient condition. The data were corrected for the sample holder, and the diamagnetic contributions were calculated from Pascal’s constants.

### 2.3 Crystal structure determination

X-ray diffraction data for compounds **1**–**3** were collected using a three-circle Bruker D8 QUEST PHOTON-III CCD diffractometer (*λ*(MoKα) radiation, graphite monochromator, and *φ* and *ω* scan modes) and corrected for absorption using the SADABS program. The data were indexed and integrated using the SAINT program. Details are given in Supplementary Table S1. The structures were solved by direct methods and refined by the full-matrix least squares technique on F2 with anisotropic displacement parameters for non-hydrogen atoms. The hydrogen atoms of the OH groups were localized in difference Fourier maps and refined isotropically with fixed-displacement parameters [*U*_iso_(H) = 1.5*U*_eq_(O)]. The other hydrogen atoms were placed in calculated positions and refined within the riding model with fixed isotropic displacement parameters [*U*_iso_(H) = 1.5*U*_eq_(C) for the CH_3_ groups and 1.2*U*_eq_(C) for the other groups]. All calculations were carried out using the SHELXTL program suite.

Crystallographic data have been deposited in the Cambridge Crystallographic Data Center, CCDC 2189837 (1), CCDC 2189838 (2), and CCDC 2189839 (3). Copies of this information may be obtained free of charge from the Director, CCDC, 12 Union Road, Cambridge CB2 1EZ, United Kingdom (Fax: +44 1223 336033; e-mail: deposit@ccdc.cam.ac.uk or www.ccdc.cam.ac.uk).

The crystal structure was determined in the Department of Structural Studies of Zelinsky Institute of Organic Chemistry, Moscow, Russia.

## 3 Results and discussion

### 3.1 Synthesis and crystal structures

**FIGURE 1:**
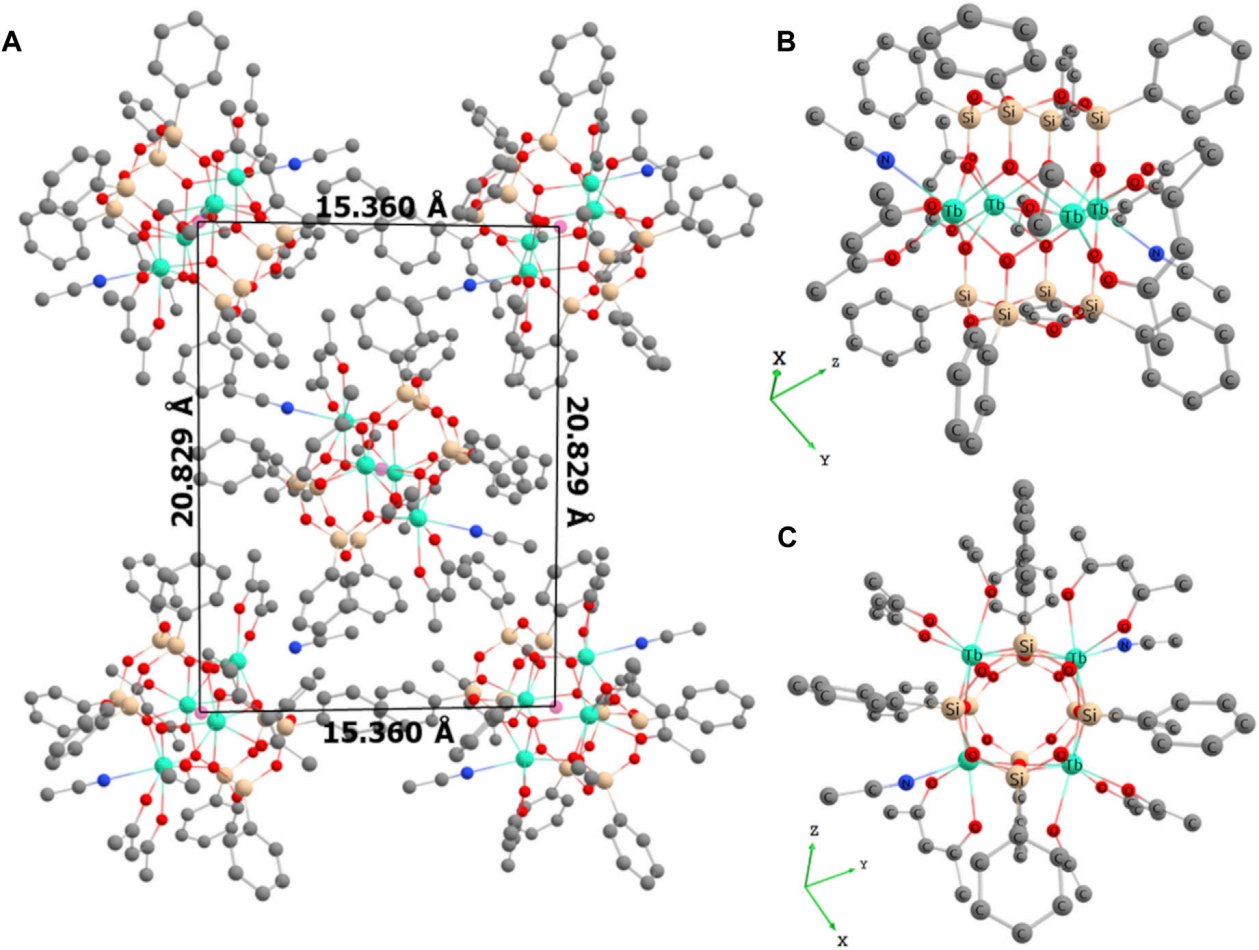
**(A)** Perspective view of crystal packing for compound **1** along the crystallographic axis *a*. Hydrogen atoms and crystallized acetonitrile molecules have been omitted for clarity; **(B)** molecular structure of compound **1** showing the prism-like polyhedron; **(C)** molecular structure of compound **1** showing the square arrangement of the Tb atoms in the (TbO_2_)_4_O core. Color code: green, Tb; beige, Si; red, O; blue, N; and gray, C.

The synthesis of Ln^3+^-based silsesquioxanes (PhSiO_1.5_)_8_(LnO_1.5_)_4_(O)(C_5_H_8_O_2_)_6_(EtOH)_2_(CH_3_CN)_2_⋅2CH_3_CN (where Ln = Tb **1**, Tb/Eu **2**, and Gd **3**) with an antenna ligand was performed using a two-step approach involving conventional alkaline hydrolysis (Prigyai et al., 2019; Laird et al., 2021) with the *in situ* formation of phenylsiloxanolate [PhSi(O)ONa]_x_ species, following a self-assembling reaction with the *acac* ligand and the corresponding Ln^3+^ salts for compounds **1** (Tb^3+^) and **3** (Gd^3+^) or mixed Tb^3+^ and Eu^3+^ salts (with a Tb^3+^/Eu^3+^ ratio of 3/1) for compound **2**. The crystallization from an acetonitrile/ethanol mixture affords the formation of single crystals suitable for crystallographic analysis. Single-crystal X-ray diffraction performed on compounds **1–3** indicates that they are isostructural and crystalize in the *P*2_1_/n space group (Supplementary Table S1, Electronic Supplementary Material (ESI)). Their crystal packing may be viewed as an assembly of neutral tetranuclear lanthanide-based cages directed almost toward [–1 1–1] (Figure 1A for compound **1**; Supplementary Figures S1,S2, ESI for compounds **2**–**3**). In all compounds, the cages form layers parallel to (1 0–1). Within the layers, the cages are arranged perpendicular to each other. Two acetonitrile molecules were also crystalized in the unit cell and situated between the tetranuclear cages. The shortest intermolecular Ln–Ln distances are equal to 10.0765, 10.0709, and 10.0981 Å for compounds **1**, **2**, and **3**, respectively.

The molecular structure of the neutral tetranuclear lanthanide-based cages of compound **1** may be described as a prism-like polyhedron in the form of a New Year paper lantern. It is formed by a (TbO_2_)_4_ core wedged between two tetraphenylcyclotetrasiloxanolate moieties (Figure 1B, C for compound **1**).

For compound **2**, the prism-like polyhedron is formed by a (Tb_1-x_Eu_x_O_2_)_4_ core, where x = 0.25, with statistically distributed Tb/Eu ions linked through oxygen atoms, forming a distorted square (Supplementary Figure S1, ESI). In both compounds, there are two slightly different seven-coordinated Tb (for compound **1**) or Tb/Eu sites (for compound **2**), which adopt a one-capped trigonal prism geometry. Each Ln^3+^ ion is coordinated by four bridging oxygens, two oxygens from terminal acetylacetonate, and one ethanol or acetonitrile molecule. The Tb (or Tb/Eu)-O distances involving bridging oxygens are in the range 2.3121(1)–2.3471(1) Å for compound 1 and 2.3013(2)–2.3789(2) Å for compound **2**, while those involving terminal acetylacetonate and ethanol molecules are larger and situated in the range 2.2884(1)–2.3966(1) Å for compound **1** and 2.2822(6)–2.4401(6) Å for compound **2**. The Tb (or Tb/Eu)-N distances involving terminal acetonitrile are 2.5358 (2) Å for compound **1** and in the range 2.4564–2.5691 Å for compound **2**. The O-Ln-O angles in the square are in the range 70.322(3)–81.654(3)° for compounds **1** and **2**. Compound **3** presents crystal structures similar to those of compound **1** (Supplementary Figure S2, ESI). The main distances and angles are given in ESI (Supplementary Table S2). The atomic Tb/Eu ratio in compound **2** determined by SEM-EDX analysis is equal to 3/1, as expected.

### 3.2 Magnetic properties

The magnetic measurements were determined for all compounds using a SQUID MPMS-XL magnetometer working between 1.8 and 300 K and up to 7 T.

**FIGURE 2:**
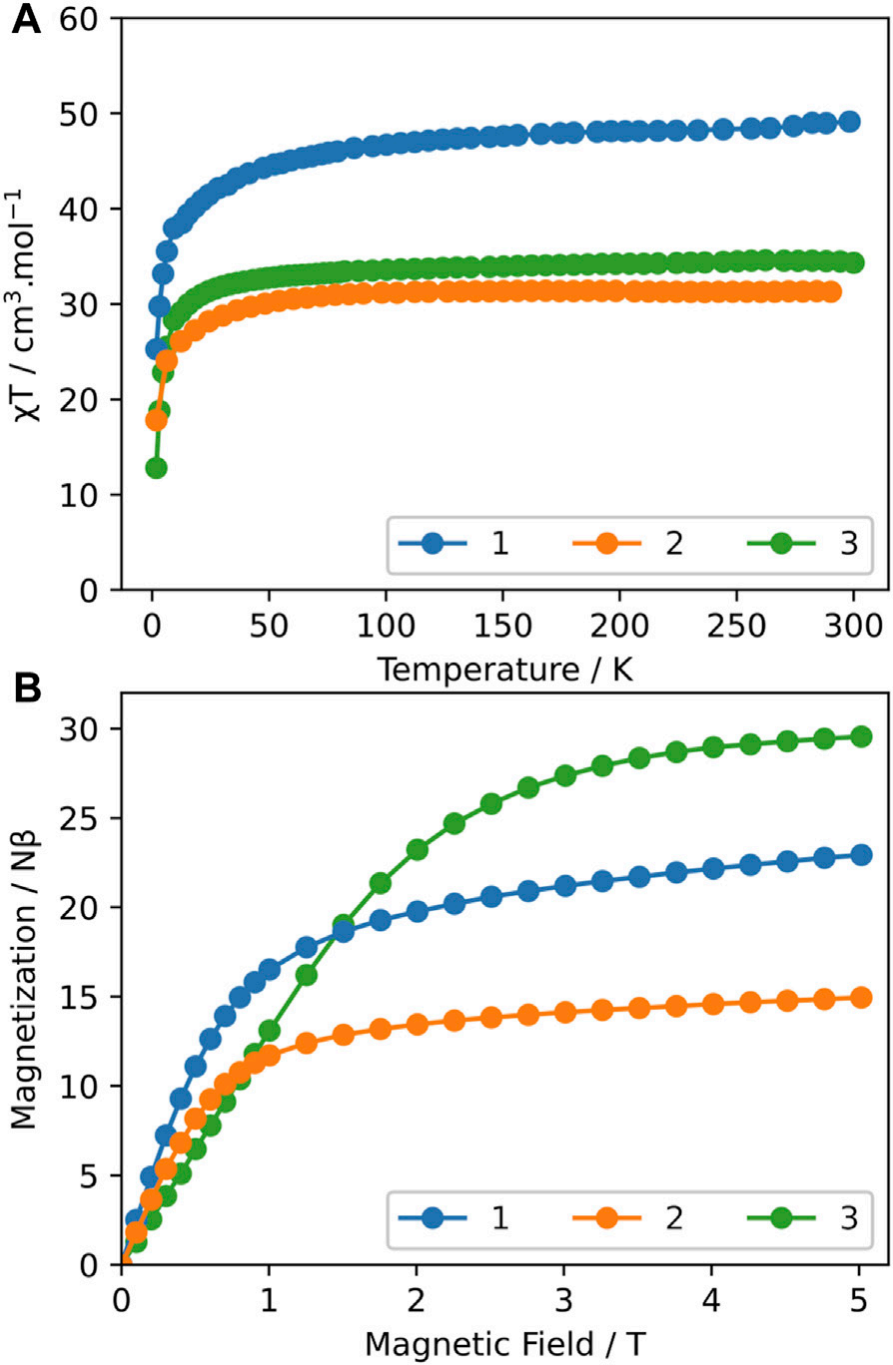
**(A)** Temperature dependence of *χT* under a 1,000-Oe dc magnetic field for compounds **1**–**3**; **(B)** field dependence of the magnetization at 1.8 K for compounds **1**–**3**.

The temperature dependence of the magnetic susceptibility performed in the direct current (dc) mode has been performed under an applied magnetic field of 1,000 Oe. The room temperature *χT* values of 48.40 and 31.30 cm^3^∙K∙mol^−1^ for compounds **1** and **2**, respectively, are coherent with the theoretical values of 47.28 and 35.46 cm^3^∙K∙mol^−1^ expected for four (compound **1**) and three Tb^3+^ ions (compound **2**), using the free-ion approximation (^7^F_6_, S = 3, L = 3, g = 3/2, *χT* = 11.82 cm^3^∙K∙mol^-1^) (Long et al., 2011). Upon cooling, the compounds exhibit a typical decrease in *χT* caused by the thermal depopulation of the Stark sublevels and/or the presence of antiferromagnetic interactions between the Tb^3+^ centers (Figure 2A). The field dependence of magnetization performed at 1.8 K shows a rapid linear increase in the magnetization with the field for both compounds (Figure 2B). The curves do not reach the saturation and magnetization values of 22.9 and 14.95 *Nβ* under 7T for compounds **1** and **2**, respectively, indicating the presence of significant magnetic anisotropy. This behavior is perfectly coherent with the previously published cage-like compounds containing Tb^3+^ ions. Note that no slow relaxation of the magnetization has been observed by investigation of the dynamic magnetic behaviors of these compounds by alternating current (ac) magnetic measurements.

The magnetic behavior of Gd^3+^-based silsesquioxane **3** is coherent with the presence of four isotrope Gd^3+^ ions. The room temperature *χT* value of 34.4 cm^3^∙K∙mol^−1^ is in agreement with the expected value (31.52 cm^3^∙K∙mol^−1^) calculated for four Gd^3+^ ions in a free-ion approximation (^8^S_7/2_, g = 2) (Long et al., 2011). As the temperature decreases, the *χT* vs*. T* curve almost remains constant up to 20 K and then sharply declines at low temperatures, indicating the presence of weak antiferromagnetic interactions between adjacent Gd^3+^ ions (Figure 2A). The M vs*.* H curve performed at 1.8 K is coherent with the presence of four Gd^3+^ ions (28 Nβ) (Figure 2B).

### 3.3 Experimental and theoretical photoluminescence studies

The excitation and emission spectra of all compounds were investigated in the solid state at different temperatures ranging from 11 to 378 K.

#### 3.3.1 Gd^3+^ compound **3**

The photoluminescence of Gd^3+^-containing compound **3** has been used to assess the energies of the triplet states located on the terminal *acac* ligand since Gd^3+^ has a high energy-accepting electronic level, which prevents any population through the energy transfer from the electronic level of the antenna ligand. Supplementary Figure S3, ESI, shows the emission spectrum of compound **3** obtained upon excitation at 246 nm at 77 K. The energy of the triplet state is 25,316 cm^−1^ (corresponding to an emission peak at 395 nm).

#### 3.3.2 Tb^3+^ compound **1**

**FIGURE 3:**
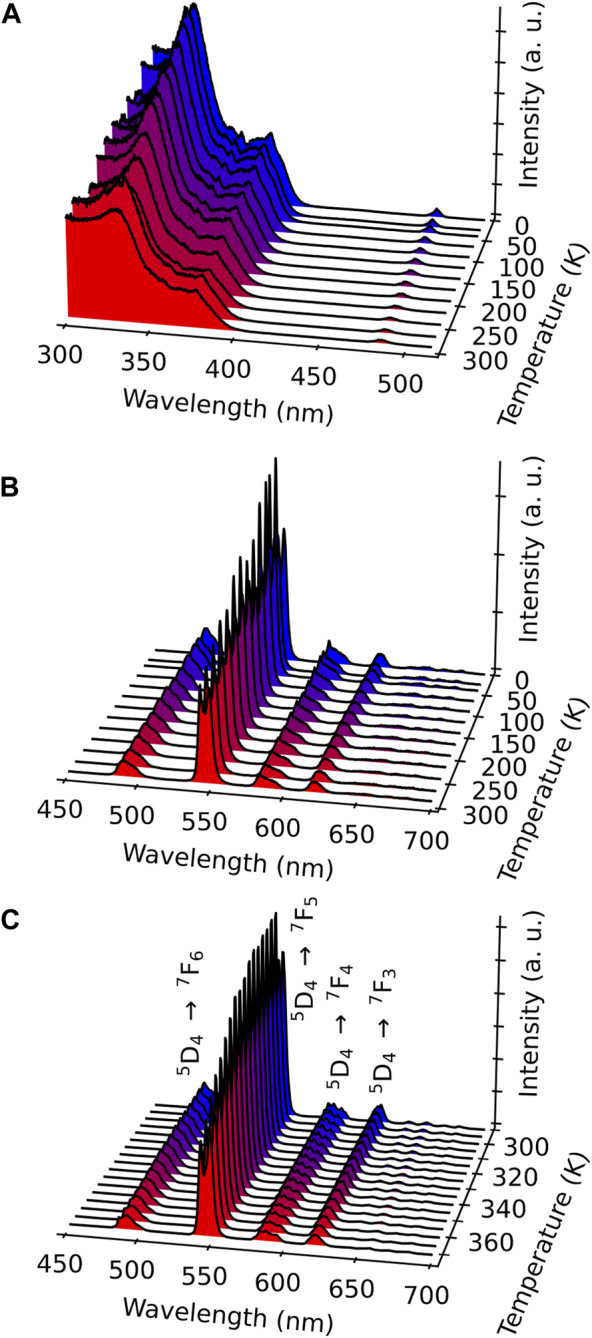
**(A)** Excitation spectra of compound **1** monitored at 543 nm in the range 11–297 K. Emission spectra of compound **1** performed with excitation at 330 nm measured from 11 to 297 K **(B)** and from 298 to 378 K **(C)**.

**FIGURE 4:**
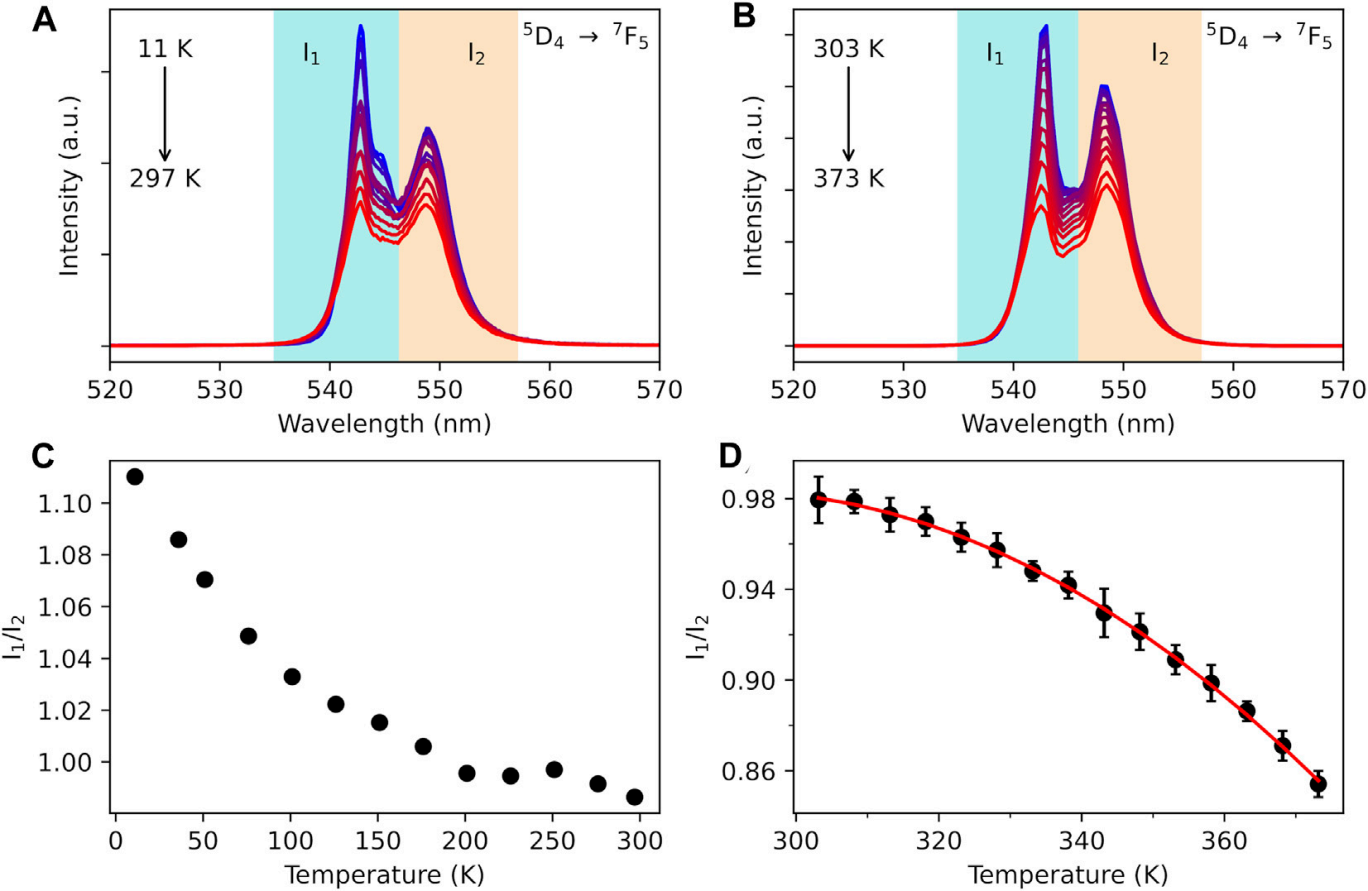
⁵D_4_→⁷F_5_ transition emission band for compound **1** upon the excitation at 330 nm in the ranges 11–297 K **(A)** and 303–378 K **(B)**. The shadowed blue and orange regions represent the integration ranges for the I_1_ and I_2_ Stark components, respectively. Temperature dependence of the I_1_/I_2_ ratio in the intervals 11–297 K **(C)** and 303–378 K **(D)**. The red curves represent a single exponential function as the best fit to the experimental data (*r* ^*2*^ > 0.99).

**FIGURE 5:**
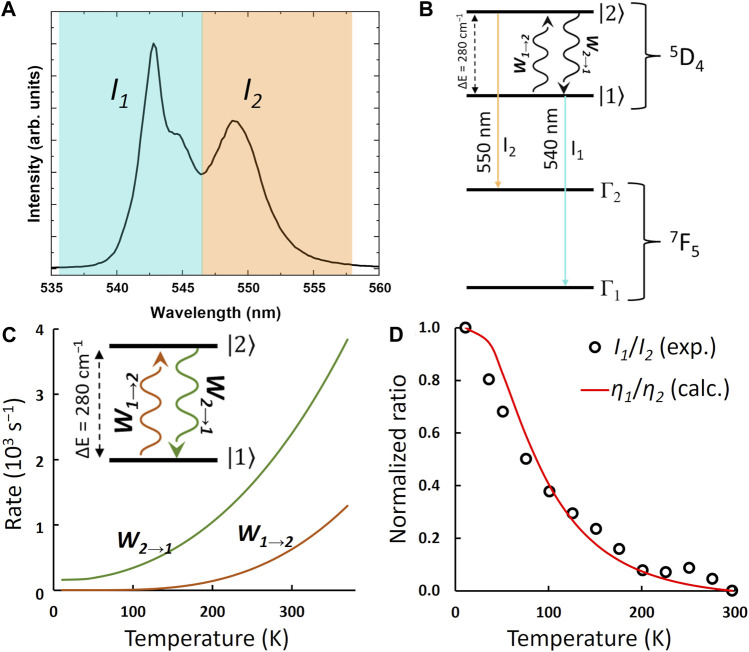
**(A)** Low-temperature (11 K) emission spectrum, recorded with excitation at 322 nm, showing the two main Stark levels of the ^5^D_4_→^7^F_5_ transition. **(B)** Illustration of the emission process between different Stark components of the ^5^D_4_ level to different Stark components of the ^7^F_5_ level. **(C)** Calculated multiphonon rates, as a function of temperature, exchanged by ^5^D_4_ Stark levels $\left|1\right.\rangle$ and $\left|2\right.\rangle$ . **(D)** Normalized intensity ratio (I_1_/I_2_) vs temperature.

The excitation spectra of Tb^3+^-containing compound **1** monitored within the main ^5^D_4_→^7^F_5_ transition (543 nm) in the range 11–300 K exhibit large broadband, with a main component at *ca*. 330 nm attributed to the *acac* ligand excited state (Figure 3A). A series of low-intensity narrow lines attributed to transitions between ^7^F_6_ and the ^5^G_5_, ^5^G_6_, and ^5^D_3_ excited states indicates that Tb^3+^ is mainly populated through antenna-assisted sensitization rather than by a direct excitation into the intra-4f^8^ lines. The emission spectra of compound **1** recorded under excitation at 330 nm in the 11–297-K and 298–378-K intervals (Figures 3B,C) exhibit the classical Tb^3+^ ^5^D_4_→^7^F_6-0_ characteristic emission lines. The profile of the emission spectra is similar under direct 4f^8^ excitation at 485 nm (Supplementary Figures S4A, B, ESI). The most intense emission, centered at 543 nm, corresponds to the ^5^D_4_→^7^F_5_ transition, displaying a gradual decrease with increasing temperature, as shown in Figure 4 and Supplementary Figures S4C, S5 (ESI). The emission decay curve of compound **1** was monitored at room temperature within ^5^D_4_→^7^F_5_. The curve is well-reproduced by a single exponential function, yielding a ^5^D_4_ lifetime of 0.851 ± 0.002 ms, which is a rather typical value for Tb^3+^ complexes (Supplementary Figure S6). The presence of two maxima in the ^5^D_4_→^7^F_5_ transition, *I*_1_ and *I*_2_ in Figure 4, is associated with two distinct Stark components. Although the maximum number of ^5^D_4_ and ^7^F_5_ Stark levels can lead to 99 components in the ^5^D_4_→^7^F_5_ transition, once the majority is degenerate, only a few can be observed (Figure 4). A question can be raised concerning whether some of these transitions between Stark levels could be attributed to vibronic transitions (or sidebands). This can be answered with the emission spectrum recorded at 11 K (Figure 5A), where vibrational modes that could couple in this spectral region can be suppressed. As the emission intensity is directly proportional to the population of the emitting level, the model suggests that emission *I*_1_ originates from a Stark component ( $\left|1\right.\rangle$ in Figure 5B) associated with the lower Stark level of ^5^D_4_. In contrast, emission *I*_2_ comprises the emission from a Stark level with higher energy ( $\left|2\right.\rangle$ in Figure 5B). It is important to note that the high-energy emission is not always attributed to a Stark level with higher energy as the energy of the transition also depends on the energy of the ending level (^7^F_5_), as shown in Figure 5B.

The model assumes that the high-energy emission stems from a lower-energy Stark component of ^5^D_4_. This assumption is relevant because, at 11 K, there would be no thermal population arising from $\left|1\right.\rangle$ to $\left|2\right.\rangle$ , and the emission *I*_2_ should vanish at low-temperature ranges because $W_{2\to 1}$ >> $W_{1\to 2}$ (Figure 5C), where $W_{2\to 1}$ and $W_{1\to 2}$ are the multiphonon decay rate (creation of phonons) and absorption (annihilation of phonons) rates, respectively, between $\left|1\right.\rangle$ and $\left|2\right.\rangle$ Stark components of the ^5^D_4_ level. The decay rate $W_{2\to 1}$ was calculated from the energy gap law (Moos, 1970; Reisfeld et al., 1977; Riseberg et al., 1977; Malkin et al., 2005) in the Miyakawa–Dexter approach (Supplementary Eqs S1,S2) (Miyakawa and Dexter, 1970). The intramolecular energy transfer (IET) rates from T_1_ to ^5^D_4_ levels were calculated using Supplementary Eqs S5–S7 (Carneiro Neto et al., 2019) (details given in ESI) using the JOYSpectra web platform (Moura Jr. et al., 2021b).

Based on the obtained IET and multiphonon rates (Figure 5C; Supplementary Table S3), together with the energy level diagram shown in Figures 5A,B, a three-level rate equation model can be considered as follows:

$$\frac{d\eta_{1}\left(t\right)}{dt}=W_{2\to 1}\eta_{2}\left(t\right)-\left(W_{1\to 2}+\frac{1}{\tau }\right)\eta_{1}\left(t\right)$$ (1)

$$\frac{d\eta_{2}\left(t\right)}{dt}=W_{1\to 2}\eta_{1}\left(t\right)+\phi \eta_{3}\left(t\right)-\left(W_{2\to 1}+\frac{1}{\tau }\right)\eta_{2}\left(t\right)$$ (2)

$$\frac{d\eta_{3}\left(t\right)}{dt}=\frac{1}{\tau }\left[\eta_{1}\left(t\right)+\eta_{2}\left(t\right)\right]-\phi \eta_{3}\left(t\right)$$ (3)

where $\eta_{1}$ , $\eta_{2}$ , and $\eta_{3}$ are the populations of $\left|1\right.\rangle$ , $\left|2\right.\rangle$ , and ^7^F_5_, respectively, with initial conditions (when t = 0) of $\eta_{1}\left(0\right)=\eta_{2}\left(0\right)=0$ and $\eta_{3}\left(0\right)=1$ . τ=0.851 ms is the measured decay lifetime of the ^5^D_4_ level, and ϕ is the feeding rate of the emitting level ^5^D_4_ that comes mainly from the energy transfer rates involving the ^7^F_6_ and ^7^F_5_ levels as the starting level (e.g., IET rates from ligand states to Tb^3+^ ^7^F_6_→^5^D_4_ and ^7^F_5_→^5^D_4_) (Kasprzycka et al., 2020; Carneiro Neto et al., 2022).

One premise of the present model is that the ϕ feeding rate is attributed to the direct energy transfer from the T_1_ state to the ^5^D_4_ level, following the pathways [T_1_→S_0_]→Tb^3+^[^7^F_6_→^5^D_4_] and [T_1_→S_0_]→Tb^3+^[^7^F_5_→^5^D_4_] (Supplementary Table S3). It is noteworthy that the last pathway dominates the direct energy transfer process with a rate of 1.9 × 10^6^ s^−1^, and the exchange mechanism (Supplementary Eq, S7) has the most significant contribution (Supplementary Table S3).

Although the energy transfer from the S_1_ state has been a recent topic of debate in the literature (Alaoui, 1995; Rodríguez-Cortiñas et al., 2002; Yang et al., 2004; Kasprzycka et al., 2017; Moura Jr. et al., 2021a; Aquino LE do et al., 2021; Manzur et al., 2023), as certain levels of Tb^3+^ may serve as better acceptors due to the high values of matrix elements involved in the IET process (e.g., ^7^F_6_→^5^G_6_ and ^7^F_5_→^5^G_6_) (Moura Jr. et al., 2021a), the population in the upper levels essentially decays not as quickly to the ^5^D_4_ level compared to the rising of the population from the ^7^F_6_ and ^7^F_5_ to the ^5^D_4_ level in the direct energy transfer process. Thus, this premise can be justified by the subsequent multiphonon decay between adjacent levels of Tb^3+^, forming a ladder-like decay process. These decays are generally slower than direct energy transfer, and consequently, decay steps like ^5^G_6_→^5^D_3_→^5^D_4_ can be neglected in the present model.

For comparison, the decay from ^5^D_3_→^5^D_4_ involves a large energy gap of Δ(^5^D_3_→^5^D_4_) ≈ 5,792 cm^−1^ (Carnall et al., 1978), leading to a decay rate of $W_{mp}$ = 7.4 × 10^3^ s^−1^ if two optical phonons with $\hbar \bar{\omega }$ = 2,896 cm^−1^ each are considered to bridge the Δ(^5^D_3_→^5^D_4_) gap. This calculation is based on the application of Supplementary Eqs S1, S2 within the energy gap law framework. If a three-phonon process is required to bridge the gap, the multiphonon rate will be even lower ( $W_{mp}$ ∼ 60 s^−1^), as expected when the number of phonons is increased. This underscores that the direct IET rates from the T_1_ state are more than two orders of magnitudes higher (see Supplementary Table S3) than the multiphonon decay in the ^5^D_4_ sensitization process.

The rate equation model (Supplementary Eqs S1–S3) was numerically propagated using the Radau method, a numerical approach belonging to the class of fully implicit Runge–Kutta methods (Hairer et al., 2015), over a time span of 0–10 ms with a step size of 10 ns. This implies that 1,000,000 points were calculated for each temperature, ranging from 11 to 297 K in steps of 2 K.

As the intensity is directly proportional to the population of the emitting level, the experimental intensity ratio ( $I_{1}/I_{2}$ in Figure 4) and the calculated population ratio ( $\eta_{1}/\eta_{2}$ ) in the steady-state regime can be compared. Figure 5D illustrates this comparison, and it can be concluded that the calculated trend is similar to the experimental trend. This observation suggests that the Stark components of the ^5^D_4_ are governed by an equilibrium between non-radiative decay and rising among these components ( $\left|1\right.\rangle$ and $\left|2\right.\rangle$ ), driven by a Boltzmann distribution between $W_{2\to 1}$ and $W_{1\to 2}$ .

Suta and Meijerink (2020) reported that there is potential temperature dependence in the use of crystal field splitting (Stark levels) for trivalent lanthanides at low temperatures. However, at higher temperatures, where the product k_B_T is significantly greater than the crystal splitting energy, a thermodynamic equilibrium among Stark levels could be established, allowing for their treatment as an effectively thermally averaged single level with an average radiative decay rate. In our model, this implies that the non-radiative decays and absorptions between two Stark levels are approximately equal, rendering the model inapplicable. In other words, Boltzmann statistics between Stark levels become ineffective at higher temperatures, resulting in a shift in the trend for temperatures exceeding 250 K, as shown in Supplementary Figure S5A (ESI), and consistent with the behavior shown in Supplementary Figure S5B (ESI).

#### 3.3.3 Mixed Eu^3+^/Tb^3+^ compound 2

**FIGURE 6:**
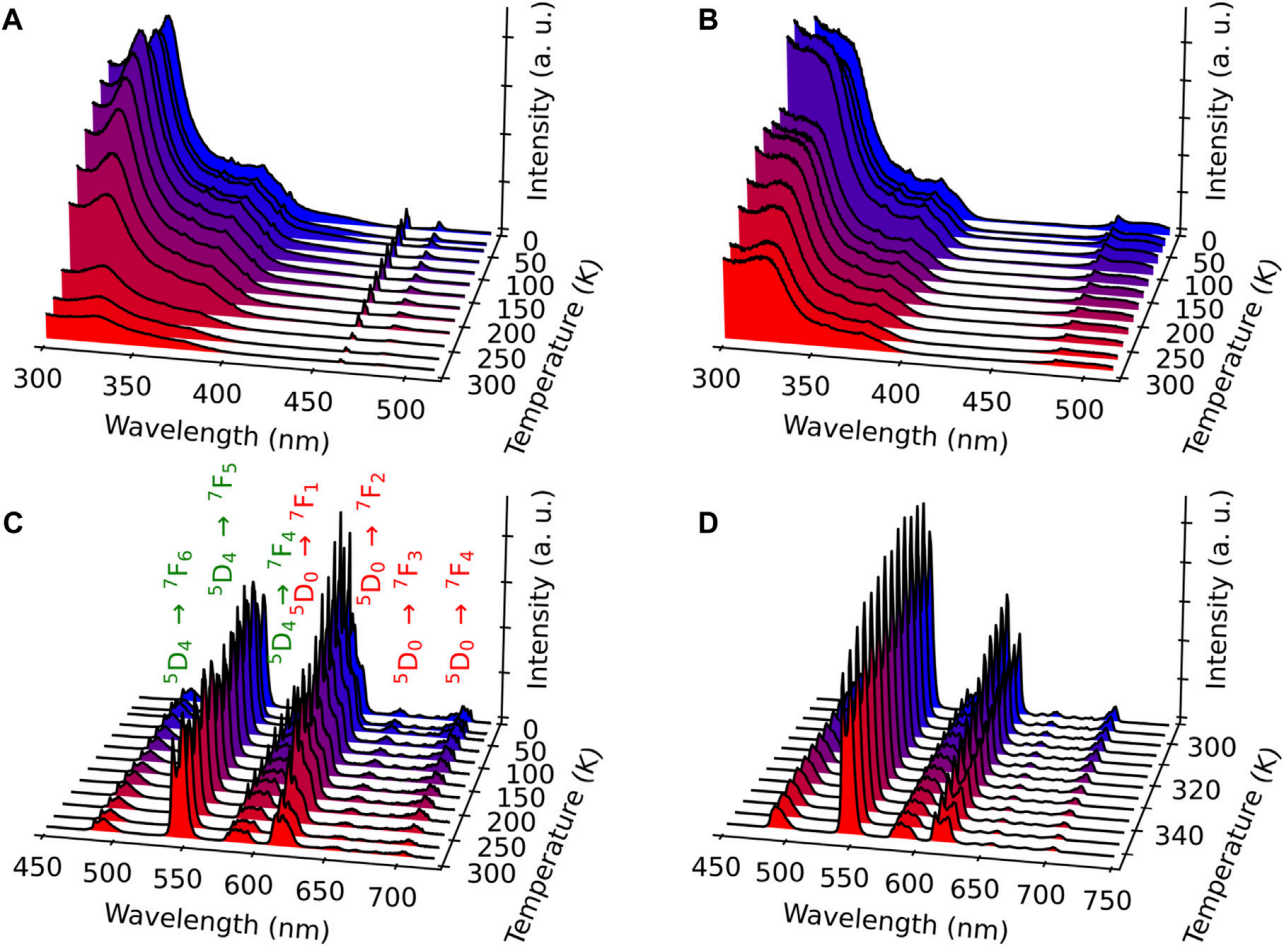
Excitation spectra of compound **2** monitored at 615 nm (Eu^3+^) **(A)** and 543 nm (Tb^3+^) **(B)** in the range 11–297 K. Emission spectra of compound **2** excited at 330 nm in the ranges 11–297 K **(C)** and 298–378 K **(D)**. The transitions ascribed to Eu^3+^ and Tb^3+^ are indicated in red and green, respectively.

To provide a self-calibrated ratiometric luminescent thermometer, the Eu^3+^ ion was introduced in the structure to obtain compound **2**, as previously demonstrated in different MOF materials and Ln^3+^-based complexes (Brites et al., 2019). The excitation spectra at different temperatures were recorded by monitoring the main emissions of both Eu^3+^ at 615 nm (^5^D_0_→^7^F_2_) and Tb^3+^ at 543 nm (^5^D_4_→^7^F_5_) (Figures 6A,B). The spectra are relatively similar and, as in the case of compound **1**, present main broadband (at 330 nm) attributed to the *acac* ligand excited states, confirming the excitation through antenna sensitization. Low-intensity intra-4f transitions of both Ln^3+^ ions can also be visible in the spectra, e.g., ^7^F_0,1_→^5^D_2_ (Figure 6A) and ^7^F_6_→^5^D_4_ (Figures 6A,B). The observation of this latter Tb^3+^ line when monitoring Eu^3+^ emission at 615 nm points out the occurrence of Tb^3+^-to-Eu^3+^ energy transfer.

The emission spectra were measured upon excitation at 330 nm, which is operational for both Ln^3+^ ions, in the ranges 11–297 K (Figure 6C) and 298–378 K (Figure 6D). Compared with the emission spectra of compound **1**, the Eu^3+^-characteristic transitions from ^5^D_0_ to ^7^F_J_ (J = 0–4) appear besides the Tb^3+^ transitions. Note that the emission spectra obtained under a direct excitation at 484 nm (Tb^3+^ ^7^F_6_→^5^D_4_ transition) show, besides the main Tb^3+^ transition ^5^D_4_→^7^F_5_ (indicated in green), a series of the Eu^3+^-related transitions ^5^D_0_→^7^F_1,2,3,4_ (indicated in red, Supplementary Figure S7, ESI). This fact points out the presence of the Tb^3+^-to-Eu^3+^ energy transfer, as already inferred from the excitation spectra shown in Figure 6B. This is not surprising considering that the shortest Ln^3+^–Ln^3+^ distance in compound **2** is equal to 3.728 Å. As expected, the emission spectra performed with direct excitation in the Eu^3+^-related band at 464 nm display only the Eu^3+^ transitions (Supplementary Figure S8, ESI).

To estimate the energy transfer rates, in addition to the calculations of pairwise interactions (Malta, 2008; Carneiro Neto et al., 2020), the distribution of donor–acceptor distances, where Tb^3+^ is the donor and Eu^3+^ is the acceptor, was calculated from the crystallographic structure using a custom program written in C. The simulations of the Tb^3+^/Eu^3+^ ratio (3:1) in the structure of an expanded 20 × 20 × 20 crystal, consisting of 64,000 Ln^3+^ sites, were conducted. The sites could be occupied by either Tb^3+^ or Eu^3+^ ions. The program performed 100 simulations while maintaining the 3:1 ratio to provide statistically reliable donor–acceptor distance results. Thus, the occurrence of the formation of a Tb-Eu pair with a given distance is given by (Trannoy et al., 2021)

$$O_{i}=\frac{N_{i}}{s∙x}$$ (4)

where $N_{i}$ is the counting of a donor–acceptor pair with distance $R\left(i\right)$ , s= 64,000 is the number of total host sites (i.e., Ln^3+^ sites) available for Eu^3+^ and Tb^3+^ substitution, and x is the fraction of Eu^3+^ ( x = 0.25 for forward energy transfer) or Tb^3+^ ( x = 0.75 for backward energy transfer).

Figure 1A and Supplementary Figure S1, ESI, show that the shortest distance between two {Ln_4_} clusters of Ln^3+^ is in the order of 15 Å. This results in a weak interaction concerning Tb-Eu energy transfer between different {Ln_4_} clusters. Thus, the Tb-Eu interaction is restricted to intra-cluster energy transfer, which can lead to highly effective energy transfer rates, as discussed in the literature (Wang et al., 2014; Calado et al., 2023; Gálico et al., 2023; Pelluau et al., 2023).

Using the calculated Tb-Eu pairwise energy transfer rates (see Supplementary Table S4 and the theoretical section in the ESI for further details) and the $O_{i}$ coefficients (Supplementary Table S5; Eq. 4) obtained from doping simulations of Tb^3+^ and Eu^3+^, the average (or effective) energy transfer rates from Tb-to-Eu can be estimated as (Trannoy et al., 2021)

$$\langle W\rangle =\sum_{i=1}^{4}{\langle W\rangle }_{i}=\left(1-x\right)x\sum_{i=1}^{4}O_{i}∙W_{i}$$ (5)

where x = 0.25 is the fraction of Eu^3+^ in the compound and $W_{i}$ represents the pairwise Tb-Eu energy transfer for the *i*th distance (Supplementary Table S5).

Supplementary Figure S9 illustrates the temperature behavior of $W_{i}$ and $\langle W\rangle$ . Although the temperature increase provides high Eu^3+^ sensitization, the emission of Eu^3+^ undergoes quenching (Figures 6C,D), probably due to high-energy phonons that may couple with the ^5^D_0_ level. Thus, although intra-cluster Tb^3+^-to-Eu^3+^ energy transfer can provide high rates in contrast to non-clustering systems (Carneiro Neto et al., 2020; Trannoy et al., 2021), there are other factors in the chemical environment around Ln^3+^ that may act as a quenching channel, in this case, selectively affecting the Eu^3+^ ion, while the decrease in the intensity of the Tb^3+^ emissions may be related to the increase in the Tb^3+^-to-Eu^3+^ rates with temperature.

**FIGURE 7:**
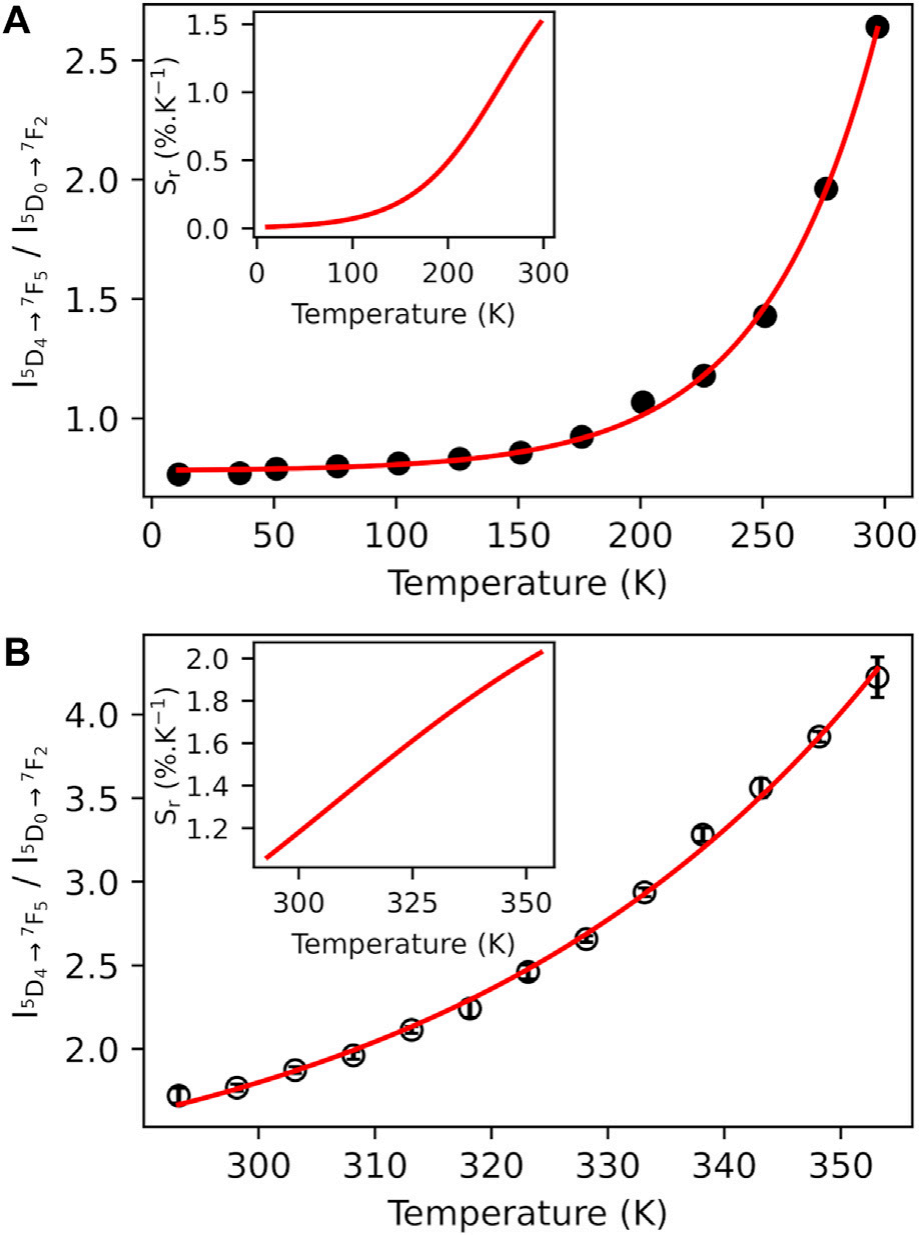
Temperature dependence of the normalized luminescence intensity (I_5D4→7F5_/I_5D0→7F2_) performed for the emission spectra of compound **2** under the excitation at 330 nm in the ranges 11–297 K **(A)** and 298–378 K **(B)** with several experimental cycles (circles) and the associated fit (red curve) with a single exponential function (full line) (*r* ^2^ > 0.99). Inset: *S*_r_, temperature dependence.

To demonstrate the possibility of using compound **2** as a self-referenced luminescent thermometer, temperature dependence of the normalized integrated intensity area related with the two main transitions Tb^3+^ ^5^D_4_→^7^F_5_ (in green) and Eu^3+^ ^5^D_0_→^7^F_2_ (in red) was extracted for both emission spectra obtained upon excitations at 330 nm (antenna) and 484 nm (intra-4f^8^). The temperature-dependent variation in the corresponding thermometric parameter (I_5D4→7F5_/I_5D0→7F2_, LIR) in the ranges 11–297 K (Figure 7A) and 298–378 K (Figure 7B) shows exponential correlations, which can be used for temperature measurements. The relative thermal sensitivity (*S*_*r*_) is the main parameter, allowing the comparison of the performance among different types of thermometers (Bednarkiewicz et al., 2020). The *S*_*r*_ value represents the variation in the experimental thermometric parameter (LIR in the present case) per degree of temperature, which is expressed as

$$S_{r}\left(T\right)=\frac{1}{LIR\left(T\right)}\left|\frac{\partial LIR\left(T\right)}{\partial T}\right|$$ (6)

The temperature dependences of *S*_*r*_ are shown in the insets of Figures 7A,B. The maximum *S*_*r*_ value is equal to 1.5%·K^−1^ at 297 K, for 11–297 K, and 2.0%·K^−1^ at 373 K, for 298–378 K. Both values are close to a frequently considered high relative thermal sensitivity (∼1%·K^−1^) (Brites et al., 2019) and are close to the best *S*_*r*_ values reported for mixed Eu^3+^/Tb^3+^ compounds (Trannoy et al., 2021). Temperature uncertainty (or thermal resolution, *δT*) is the smallest temperature change that can be detected (Brites et al., 2016). This value is related to *S*_*r*_ as follows:

$$\delta T=\frac{1}{S_{r}\left(T\right)}\left|\frac{\delta LIR\left(T\right)}{LIR\left(T\right)}\right|$$ (7)

where *δ*LIR(T) is the standard deviation in the LIR(T) obtained upon several temperature cycles. According to this, the minimal thermal resolution is 0.2 K. Note that the use of emission spectra upon excitation at 484 nm for thermometry is also possible. Supplementary Figures S10A, B, ESI show the corresponding temperature dependences of LIR and the maximal value of *S*_*r*_ of 1.3%·K^−1^ at 297 K. Therefore, the temperature sensor **2** proposed here works reliably in the operating range 11─378 K.

The emission decay curves of compound **2** were monitored at room temperature upon excitation at 330 nm within the ^5^D_4_→^7^F_6_ (Tb^3+^) and ⁵D_0_→⁷F_4_ (Eu^3+^) transitions (Supplementary Figure S11, ESI). The curves are well-reproduced by double-exponential functions as the best fits to the experimental data (*r* ^2^ > 0.99), yielding lifetimes of 0.126 ± 0.002 and 0.668 ± 0.002 ms for the former and 0.110 ± 0.002 and 0.406 ± 0.002 ms for the latter transitions. The occurrence of two lifetimes is under investigation and will be addressed later.

## 4 Conclusion

In summary, here, we reported three new luminescent cage-like silsesquioxanes containing a tetranuclear Tb^3+^, Tb^3+^/Eu^3+^, or Gd^3+^ core, where each Ln^3+^ ion is coordinated by an antenna acetylacetonate ligand. The crystal structures indicate that the introduction of chelating acetylacetonate changes the coordination environment of the Ln^3+^ ions conducting to seven-coordination geometry close to the one-capped trigonal prism.

Lanthanide-based silsesquioxanes **1** and **2** present solid-state characteristic Tb^3+^ and Tb^3+^/Eu^3+^-related emissions between 11 and 373 K sensitized by acetylacetonate antenna upon excitation in the UV region, while the direct excitation in the visible domain is also possible.

Theoretical calculations were conducted to elucidate the primary characteristics of the thermal behavior between the Stark levels of the ^5^D_4_ level in compound **1**. The results demonstrated good agreement between experiment and theory, enabling the extraction of the main effect, which is the balance between multiphonon decays and absorptions between these two Stark levels as a function of temperature.

For compound **2**, where intra-cluster energy transfer can be highly efficient and even surpass the ligand-to-Ln^3+^ transfer, simulations were performed to obtain the average Tb^3+^-to-Eu^3+^ energy transfer rates within the {Ln_4_} cluster structure. The analysis revealed that the average energy transfer increases with temperature, consistent with the low quenching observed for Tb^3+^ emissions. Conversely, the abrupt quenching of Eu^3+^ emissions with increasing temperature suggests a strong electron–phonon coupling for this ion.

Mixed Tb^3+^/Eu^3+^ (Tb/Eu ration 3/1) compound **2** exhibits a tunable thermosensitive Tb^3+^-to-Eu^3+^ energy transfer driven by Tb^3+^ and Eu^3+^ emissions. The corresponding temperature dependence allows the verification of the use of this compound for a ratiometric self-reference luminescent thermometer. It was realized by using a fluorescence intensity ratio between the two main components of the spectra (Tb^3+^ ^5^D_4_→^7^F_5_ and Eu^3+^ ^5^D_0_→^7^F_2_ transitions) in the range 11–373 K, demonstrating the maximum relative thermal sensitivity referred above 2.0 % K^−1^ at 373 K.

## Data Availability

The original contributions presented in the study are included in the article/Supplementary Material; further inquiries can be directed to the corresponding authors.
