# Supplementary material for: Luminescent Ln3+-based silsesquioxanes with a β-diketonate antenna ligand: toward the design of efficient temperature sensors
Source: Front Chem. 2024 Apr 3;12:1379587. doi: 10.3389/fchem.2024.1379587 (PMC11022212; doi:10.3389/fchem.2024.1379587)
Supplement: Supplementary file 1 [file DataSheet1.pdf]

Supplementary Material

1 Theoretical

1.1 Multiphonon rates between Stark levels

The decay rate $W_{2\rightarrow 1}$ (see Fig. 5b) can be estimated from the energy gap law [1–4] as,

$$W_{2\rightarrow 1} = W_0 e^{-(\alpha \cdot \Delta E_{21})} \tag{S1}$$

where $\Delta E_{21} = 280$ cm^{-1} is the energy difference between $|1\rangle$ and $|2\rangle$, $W_0 = 10^7$ s^{-1} is the decay rate extrapolated to zero energy gap ($\Delta E \approx 0$). The quantity α (in cm) depends on the material, and particularly on the mean phonon energy $\hbar\varpi$. According to Miyakawa-Dexter approach [5], this factor can be estimated by

$$\alpha = \frac{1}{\hbar\varpi}\left[\ln\left(\frac{N}{S[n(T)+1]}\right) - 1\right] \tag{S2}$$

where N $(= \Delta E_{21}/\hbar\varpi)$ is the number of thermally generated phonons, S is the Huang-Rhys factor whose typical values are low for trivalent lanthanides ions (in the order of 10^{-2} to 10^{-1}) [6–8]. $n(T)$ is the thermally averaged phonon occupancy number, given by a Bose-Einstein distribution:

$$n(T) = \frac{1}{e^{\frac{\hbar\varpi}{k_B T}} - 1} \tag{S3}$$

In our calculations, we adopted the values of $S = 0.02$ and $\hbar\varpi = 100$ cm^{-1}. For the reabsorption rate $|1\rangle \rightarrow |2\rangle$, a Boltzmann energy barrier from the $W_{2\rightarrow 1}$ (Eq. 1) can be used [9,10]:

$$W_{1\rightarrow 2} = W_{2\rightarrow 1} e^{-\left(\frac{\Delta E_{21}}{k_B T}\right)} \tag{S4}$$

where k_B is the Boltzmann constant and T is the temperature.

1.2 Ligand-to-Tb^{3+} energy transfer rates

The intramolecular energy transfer (IET) rates from ligands to the Tb^{3+} were calculated considering the dipole–dipole (W_{d-d}), dipole–multipole (W_{d-m}), and exchange (W_{ex}) mechanisms [11–15]

$$W_{d-d} = \frac{S_L(1-\sigma_1)^2}{(2J+1)G}\frac{4\pi}{\hbar}\frac{e^2}{R_L^6}\sum_{\lambda}\Omega_{\lambda}^{FED}\langle\psi'J'\|U^{(\lambda)}\|\psi J\rangle^2 F \tag{S5}$$

$$W_{d-m} = \frac{S_L}{(2J+1)G}\frac{2\pi e^2}{\hbar} \times \sum_{\lambda}(\lambda+1)\frac{\langle r^{\lambda}\rangle^2}{\left(R_L^{\lambda+2}\right)^2}\langle f\|C^{(\lambda)}\|f\rangle^2(1-\sigma_{\lambda})^2\langle\psi'J'\|U^{(\lambda)}\|\psi J\rangle^2 F \tag{S6}$$

$$W_{ex} = \frac{(1-\sigma_0)^2}{(2J+1)G}\frac{8\pi}{\hbar}\frac{e^2}{R_L^4}\langle\psi'J'\|S\|\psi J\rangle^2 \sum_m \left|\langle\phi\right|\textstyle\sum_j \mu_z(j) s_m(j)\left|\phi^*\rangle\right|^2 F \tag{S7}$$

where R_L = 5.19 Å is the donor-acceptor distance, Ω_λ^{FED} are the intensity parameters associated with the forced electric dipole mechanism, calculated from the Simple Overlap model[16,17] through the JOYSpectra program[18] (Ω_λ^{FED} = 0.16, 0.25, and 0.37×10^{-20} cm^2 for λ = 2, 4, and 6, respectively). The values of the squared reduced matrix elements $\langle\psi'J'\|U^{(\lambda)}\|\psi J\rangle^2$ were taken from Carnall *et al.* [19] S_L is the dipole strength of the ligand transition involved in IET ($\sim 10^{-40}\ (esu)^2 \cdot cm^2$ for T_1)[15], $\langle r^\lambda\rangle$ are the 4*f* radial integrals,[20] G is the ligand state degeneracy ($G =\ 3$ for T_1), $\langle f\|C^{(\lambda)}\|f\rangle$ is the reduced matrix element of Racah's tensor operators, which assumes values of −1.366, 1.128, and −1.270 for λ = 2, 4, and 6, respectively. The shielding factors $(1-\sigma_\lambda)$ were obtained from the relation with the overlap integrals between valence orbitals of the pair Ln–X (X is the ligating atom in the first coordination sphere) [14,21].

In Eq. S7, s_m is the spin operator in the ligand and μ_z is the dipole operator (its z-component), the value of the element matrix of these coupled operators is $\sim 10^{-36}\ (esu)^2 \cdot cm^2$ [15,22]. The $\langle\psi'J'\|S\|\psi J\rangle$ is the reduced matrix elements of the spin operator, which were calculated using free-ion wavefunctions in the intermediate coupling scheme [23,24]. The F term in Eqs. S5–S7 is the spectral overlap factor that considers the energy mismatch condition between donor and acceptor states [11,15]. For the case of ligand-to-metal energy transfer, F can be estimated by:

$$F = \frac{G(\Delta,T)}{\hbar\gamma_L}\sqrt{\frac{\ln(2)}{\pi}}\, e^{-\left(\frac{\Delta}{\hbar\gamma_L}\right)^2 \ln(2)} \tag{S8}$$

where Δ is the band maximum energy difference between the donor state and lanthanide ion acceptor state, $\Delta = E_D - E_{Ln}$. γ_L is the bandwidth at half-height for the donor state, assumed here a typical value of $\gamma_L = 4000$ cm^{-1} for both S_1 and T_1 states. $G(\Delta,T) = exp(\Delta/k_BT)$ if Δ is negative and $G(\Delta,T) = 1$ if $\Delta \geq 0$, where k_B is the Boltzmann constant and T is the temperature.

Then, the energy transfer rates (W) are calculated by the sum over Eqs. S5–S7 in the same pathway:

$$W = W_{d-d} + W_{d-m} + W_{ex} \tag{S9}$$

Having justified that the dominant process for energy transfer to sensitize Tb^{3+} is through S_1, which feeds $^5D_4$ only indirectly via successive multiphonon decays (vide manuscript), it is important to note that these multiphonon decays are orders of magnitude lower than the direct $T_1 \rightarrow {}^5D_4$ sensitization. Therefore, Table S3 provides a summary of the rates for compound **1**.

1.3 Tb-to-Eu energy transfer rates

In this section, a theoretical procedure is presented to estimate the Tb-to-Eu energy transfer rates based on crystallographic structure and the theory of nonradiative energy transfer between lanthanide ions.[14,25] Utilizing crystallographic data allows for the determination of the arrangement of host sites that can be occupied by Eu^{3+} (acceptor) or Tb^{3+} (donor) ions.

The pairwise energy transfer rates between lanthanide ions were calculated, considering the dipole-dipole (W_{d-d}), dipole–quadrupole (W_{d-q}), quadrupole–quadrupole (W_{q-q}), exchange (W_{ex}), and magnetic dipole–magnetic dipole (W_{md-md}) mechanisms,[26,27] according to Eqs. S10–S14, respectively:

$$W_{d-d} = \frac{(1-\sigma_1^D)^2(1-\sigma_1^A)^2}{(2J_D^*+1)(2J_A+1)}\frac{4\pi}{3\hbar}\frac{e^4}{R^6}\left(\sum_\lambda \Omega_\lambda^D \langle\psi_D J_D\|U^{(\lambda)}\|\psi_D^* J_D^*\rangle^2\right)\left(\sum_\lambda \Omega_\lambda^A \langle\psi_A^* J_A^*\|U^{(\lambda)}\|\psi_A J_A\rangle^2\right)F \quad \text{(S10)}$$

$$\begin{aligned} W_{d-q,\ q-d} = {} & \frac{\left(1-\sigma_1^{D,A}\right)^2\left(1-\sigma_2^{A,D}\right)^2}{(2J_D^*+1)(2J_A+1)}\frac{\pi}{\hbar}\frac{e^4}{R^8}\langle f\|C^{(2)}\|f\rangle^2 \\ & \times\left[\left(\sum_\lambda \Omega_\lambda^D \langle\psi_D J_D\|U^{(\lambda)}\|\psi_D^* J_D^*\rangle^2\right)\langle r^2\rangle_A^2\langle\psi_A^* J_A^*\|U^{(2)}\|\psi_A J_A\rangle^2\right. \\ & \left. + \left(\sum_\lambda \Omega_\lambda^A \langle\psi_A^* J_A^*\|U^{(\lambda)}\|\psi_A J_A\rangle^2\right)\langle r^2\rangle_D^2\langle\psi_D^* J_D^*\|U^{(2)}\|\psi_D J_D\rangle^2\right]F \end{aligned} \quad \text{(S11)}$$

$$\begin{aligned} W_{q-q} = {} & \frac{(1-\sigma_2^D)^2(1-\sigma_2^A)^2}{(2J_D^*+1)(2J_A+1)}\frac{28\pi}{5\hbar}\frac{e^4}{R^{10}}\langle r^2\rangle_D^2\langle r^2\rangle_A^2\langle f\|C^{(2)}\|f\rangle^4 \\ & \times\langle\psi_D J_D\|U^{(2)}\|\psi_D^* J_D^*\rangle^2\langle\psi_A^* J_A^*\|U^{(2)}\|\psi_A J_A\rangle^2 F \end{aligned} \quad \text{(S12)}$$

$$W_{ex} = \frac{2\pi}{\hbar}\left[\left(\frac{e^2}{R}\right)\rho_{f-f}^2\right]^2 F \quad \text{(S13)}$$

$$W_{md-md} = \frac{(1-\sigma_1^D)^2(1-\sigma_1^A)^2}{(2J_D^*+1)(2J_A+1)}\frac{4\pi}{3\hbar}\frac{{\mu_B}^4}{R^6}\langle\psi_D J_D\|L+2S\|\psi_D^* J_D^*\rangle^2\langle\psi_A^* J_A^*\|L+2S\|\psi_A J_A\rangle^2 F \quad \text{(S14)}$$

where the intensity parameters Ω_λ are those obtained using the FED contribution, likewise in the Ligand-Ln^{3+} energy transfer procedure. The sets of Ω_λ (FED) values obtained, in units of 10^{-20} cm^2, are: Tb^{3+} [$\Omega_2 = 0.16$; $\Omega_4 = 0.25$; $\Omega_6 = 0.37$] and Eu^{3+} [$\Omega_2 = 0.29$; $\Omega_4 = 0.46$; $\Omega_6 = 0.72$]. The W_{q-q}, W_{ex} and W_{md-md} mechanisms are independent of the intensity parameters.

In Eq. S13, ρ_{f-f} represents the overlap integral between 4f subshells of the donor and acceptor lanthanide ions. The values of ρ_{f-f} as a function of the Tb–Eu distance (R_L) were obtained using the parametric function $\rho_{f-f}(R) = exp(a + bR + cR^2)$, with a=-0.032, b=-0.261, and c=-0.34.[21] The ρ_{f-f} decreases rapidly to zero with the increase of the donor-acceptor distance R, as demonstrated in Reference[26] for the case of Tb–Eu. This is why the W_{ex} term can often be neglected in the Ln–Ln energy transfer processes, where the donor-acceptor distances are typically higher than 4 Å,[14] in contrast to the case of intramolecular energy transfer processes in lanthanide chelates.[14,15]

In all equations related to Ln-Ln energy transfer mechanisms, the spectral overlap factor (F) is involved. This quantity is associated with the energy mismatch conditions between the donor and acceptor states, and the following expression for F has been utilized:[14]

$$F = \frac{\ln(2)}{\sqrt{\pi}} \frac{1}{\hbar^2 \gamma_{Tb} \gamma_{Eu}} \left\{ \left[\left(\frac{1}{\hbar \gamma_{Tb}} \right)^2 + \left(\frac{1}{\hbar \gamma_{Eu}} \right)^2 \right] \ln(2) \right\}^{-\frac{1}{2}} \times \exp\left[\frac{1}{4} \frac{\left(\frac{2\,\Delta E}{(\hbar \gamma_{Tb})^2} \ln 2 \right)^2}{\left[\left(\frac{1}{\hbar \gamma_{Eu}} \right)^2 + \left(\frac{1}{\hbar \gamma_{Tb}} \right)^2 \right] \ln 2} - \left(\frac{\Delta}{\hbar \gamma_{Tb}} \right)^2 \ln(2) \right] \quad \text{(S15)}$$

where $\hbar\gamma_{Tb}$ and $\hbar\gamma_{Eu}$ correspond to the bandwidths at half-height of the Tb^{3+} (donor) and Eu^{3+} (acceptor) transitions, respectively. ΔE is the energy difference between donor and acceptor transitions ($\Delta E = E_D - E_A$). In the present work, $\hbar\gamma_{Tb} = \hbar\gamma_{Eu} = 250$ cm^{-1} is considered, a value deemed acceptable concerning the nature of 4f transitions.

The energy transfer pathways were chosen with the combination of four donor states (Tb^{3+}:$^5D_4 \rightarrow {}^7F_{3\text{-}6}$) and nine acceptor states (Eu^{3+}: $^7F_{0,1,2} \rightarrow {}^5D_{0,1,2}$). The selection rules on the J quantum numbers were also taken into account: $|J - J'| \leq \lambda \leq J + J'$ for multipolar mechanisms (W_{d-d}, W_{d-q}, and W_{q-q}); $\Delta J = 0, \pm 1$ for the magnetic dipole-magnetic dipole mechanism (W_{md-md}). No defined selection rules on J appear for the exchange mechanism (W_{ex}).

For each pathway, the energy transfer rates were calculated by the sum over Eqs. S10–S14 ($\omega = W_{d-d} + W_{d-q} + W_{q-q} + W_{ex} + W_{md-md}$) and all results are shown in Table S4.

2 Figures

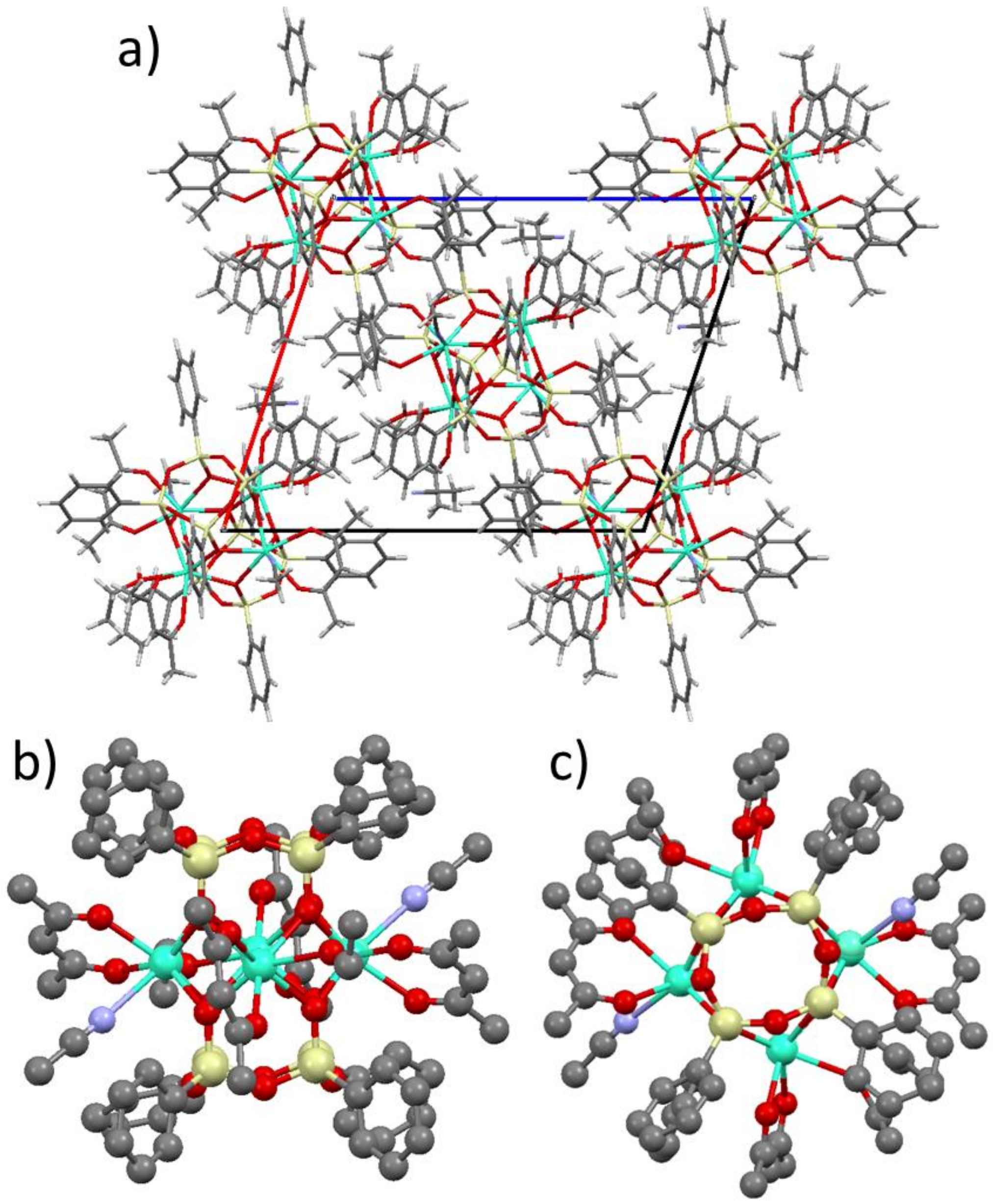

Figure S1. a) Perspective view of the crystal packing for **2** along the crystallographic axis b. Hydrogen atoms and solvate acetonitrile molecules have been omitted for clarity; b) Molecular structure of **2** showing the prism-like polyhedron in the form of a New Year paper lantern; c) Molecular structure of **2** showing the square arrangement of the Tb/Eu atoms in the $[(Tb/EuO_2)_4]$-core. Colour code: green Tb/Eu; yellow Si; red O; blue N; grey C.

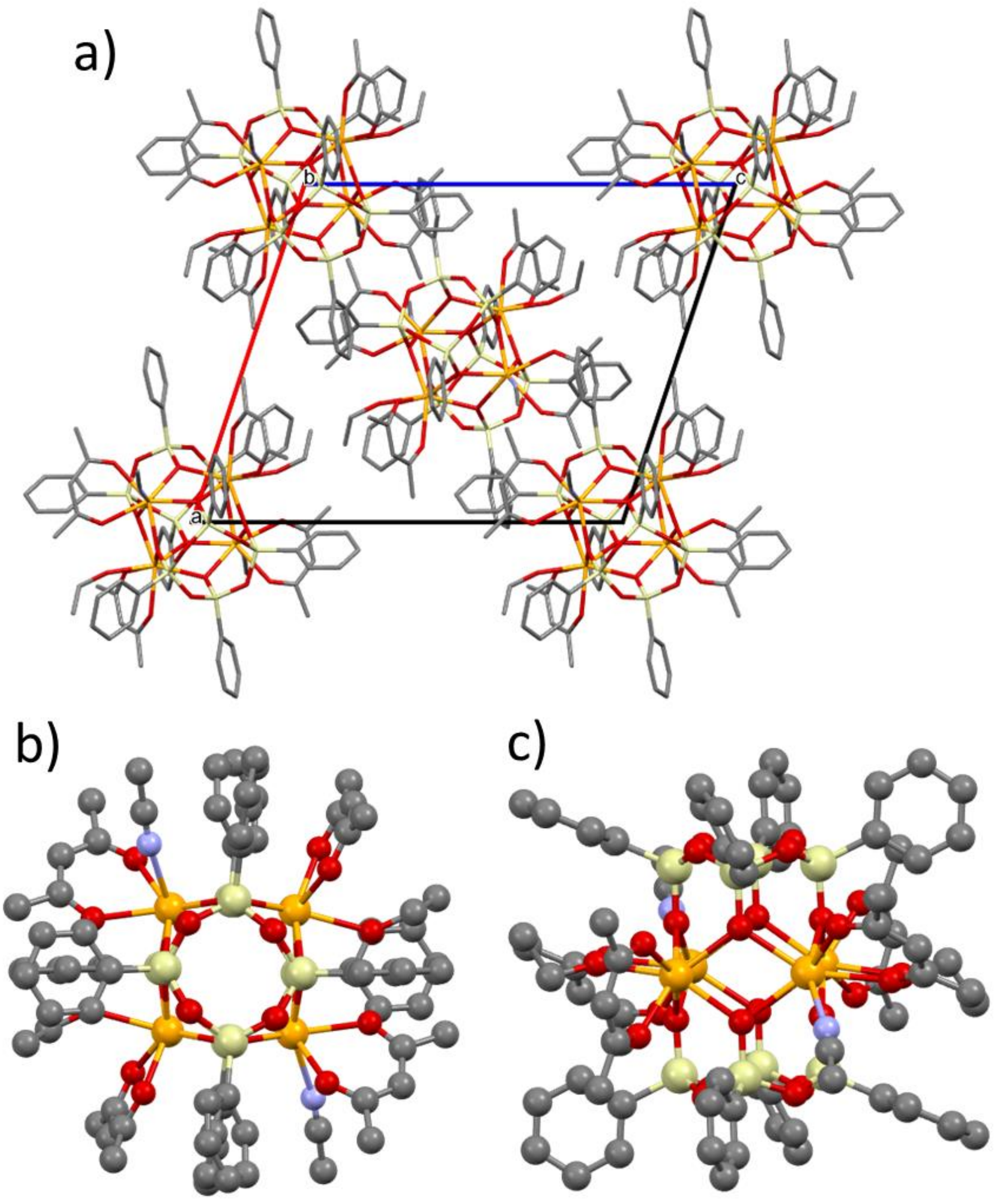

Figure S2. a) Perspective view of the crystal packing for **3** along the crystallographic axis b. Hydrogen atoms and solvate acetonitrile molecules have been omitted for clarity; b) Molecular structure of **3** showing the square arrangement of the Gd atoms in the $[(GdO_2)_4]$-core; c) Molecular structure of **3** showing the prism-like polyhedron in the form of a new year paper lantern. Colour code: orange Gd; yellow Si; red O; blue N; grey C.

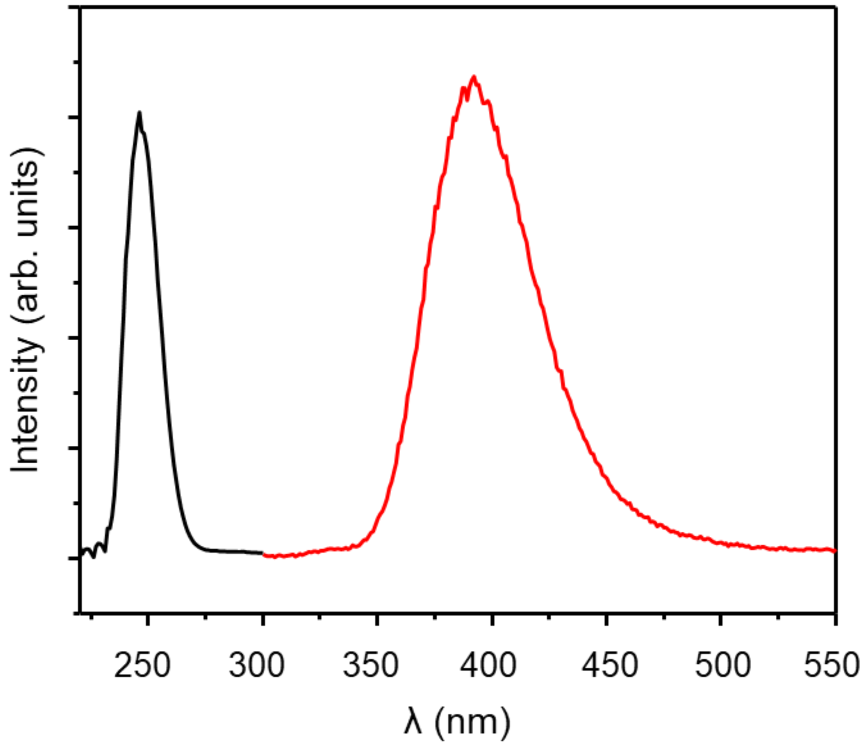

Figure S3. Excitation spectrum (black curve) monitored at 393 nm and emission spectrum (red curve) performed under excitation at 246 nm in solid state at 77 K for **3**.

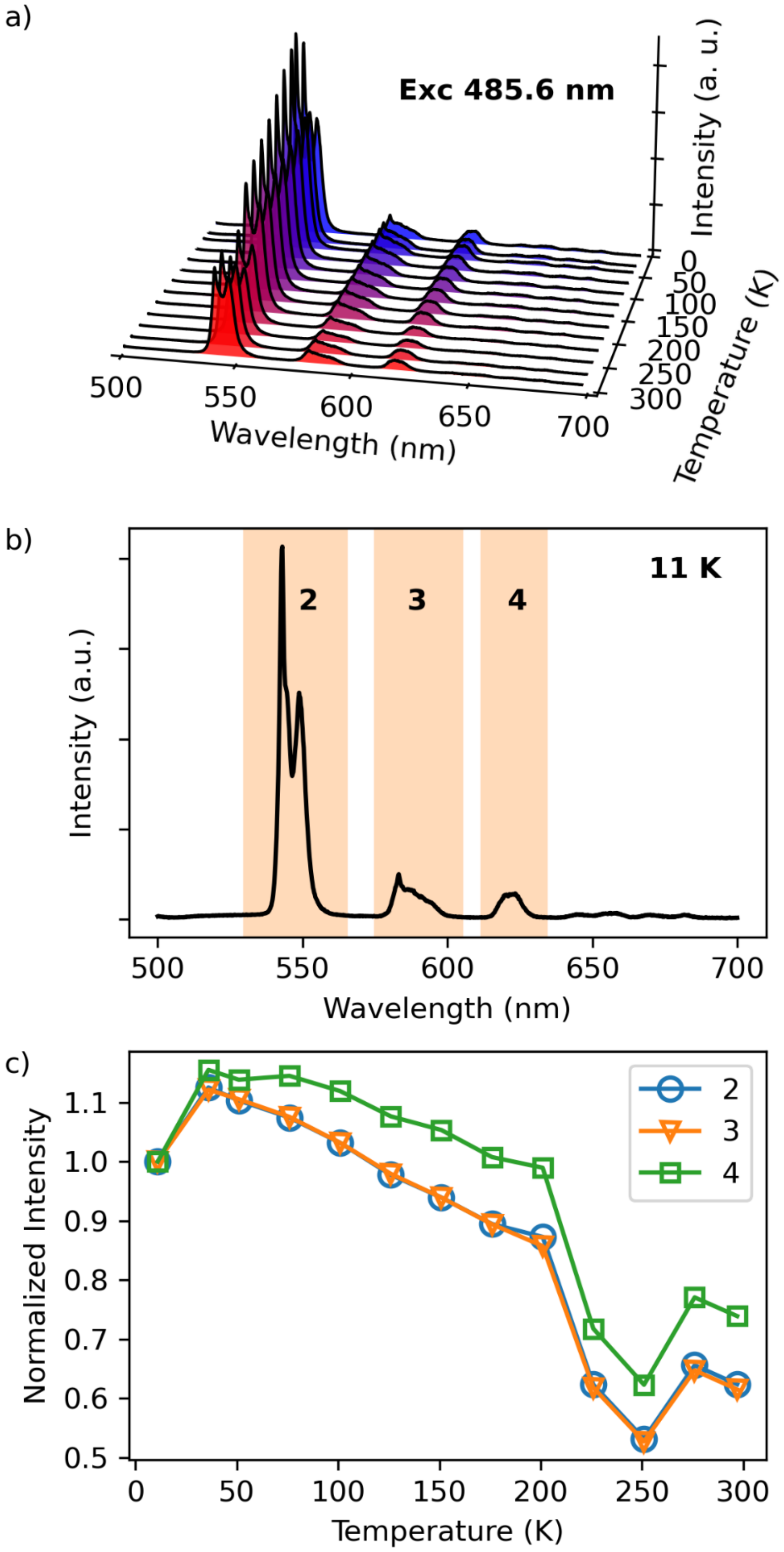

Figure S4. a) Emission spectrum of **1** upon 485.6 nm excitation performed in the 11 – 297 K temperature range; b) Emission spectrum of **1** upon 485.6 nm excitation at 11 K. The shadowed regions represent the integration ranges for each transition; c) Normalized integrated intensity area of bands indicated as 2, 3 and 4 as a function of temperature.

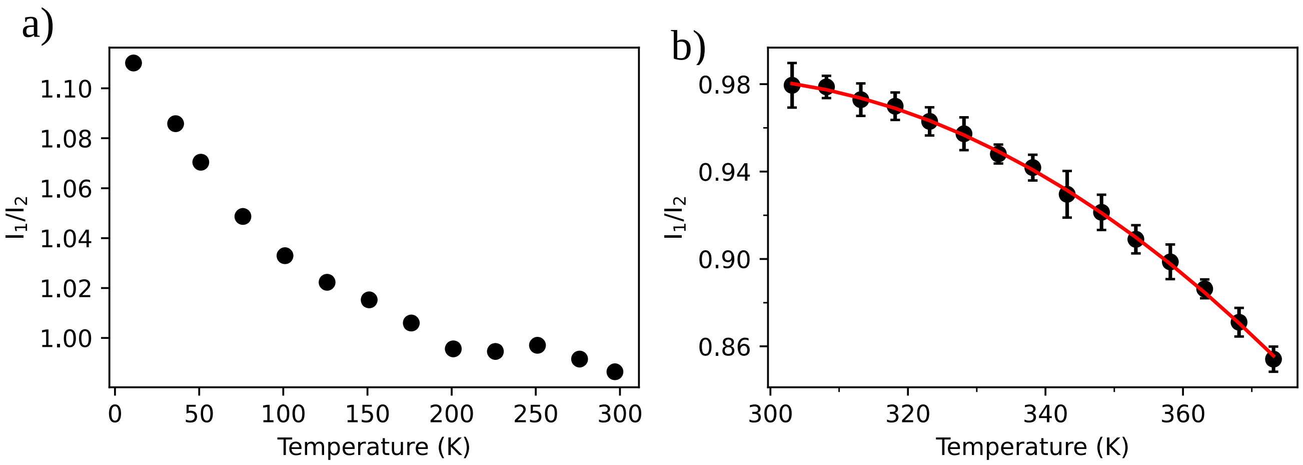

Figure S5. Temperature dependence of the I_1/I_2 ratio (the I_1 and I_2 correspond to the Starks components indicated in Figure 4) performed for the $^5D_4 \rightarrow ^7F_5$ transition emission band for **1** upon the excitation at 330 nm in the: 11 – 297 K (c) and 298 – 378 K (d) intervals. The red curves represent a single exponential function as the best fit to the experimental data ($r^2 > 0.99$).

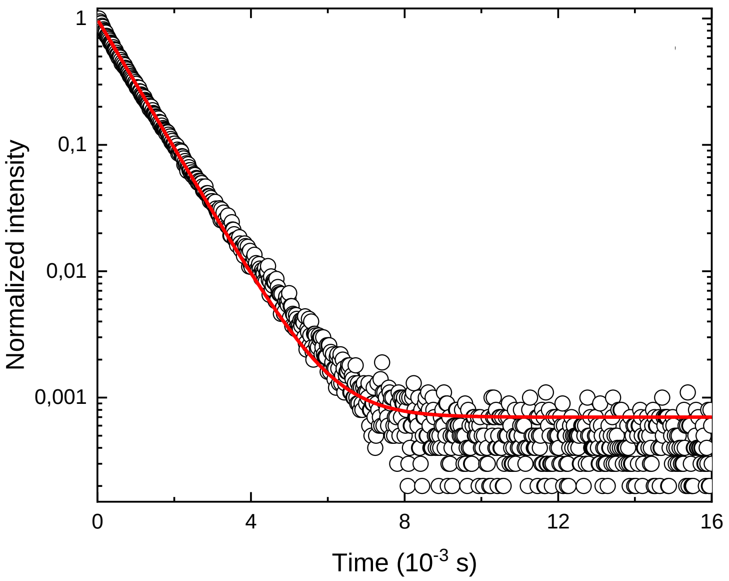

Figure S6. Temporal decay trace of **1** monitoring at 543 nm upon 330 nm excitation recorded at room temperature. The red solid curve represents a single-exponential function as the best fit to the experimental data (r>0.99).

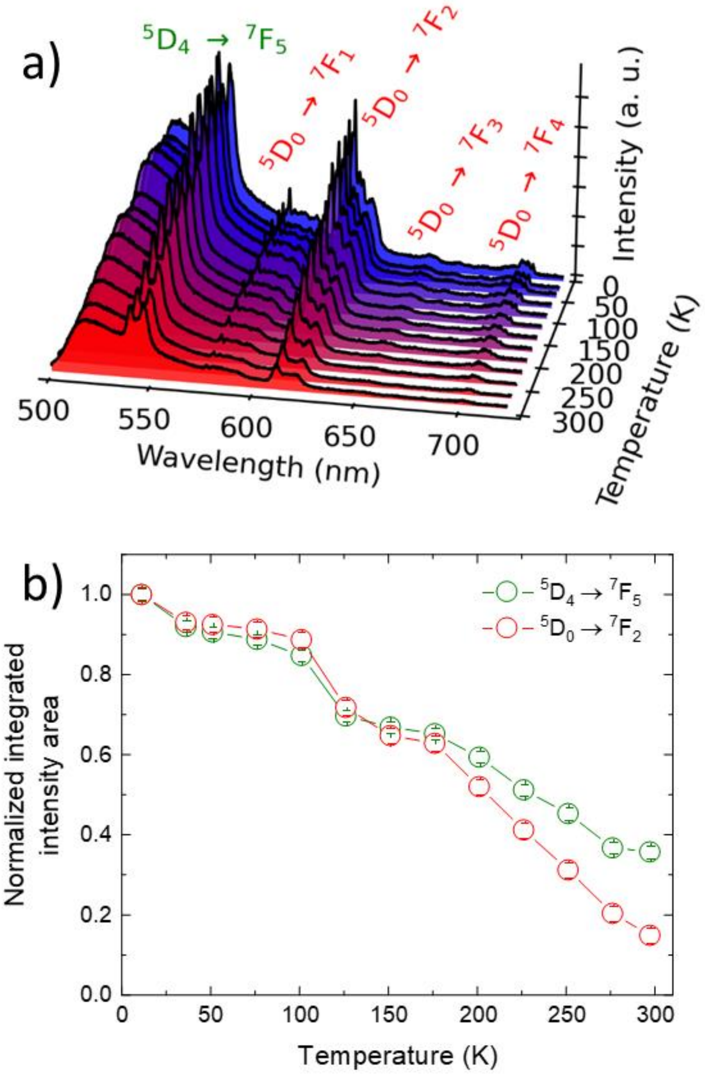

Figure S7. a) Emission spectra performed with the excitation at 484 nm in solid state in the temperature range 11 – 300 K for **2**; b) Normalized integrated intensity area of $^5D_4 - {}^7F_5$ (in green) and $^5D_0 - {}^7F_2$ (in red) transitions within the temperature range from 11 K to 300 K.

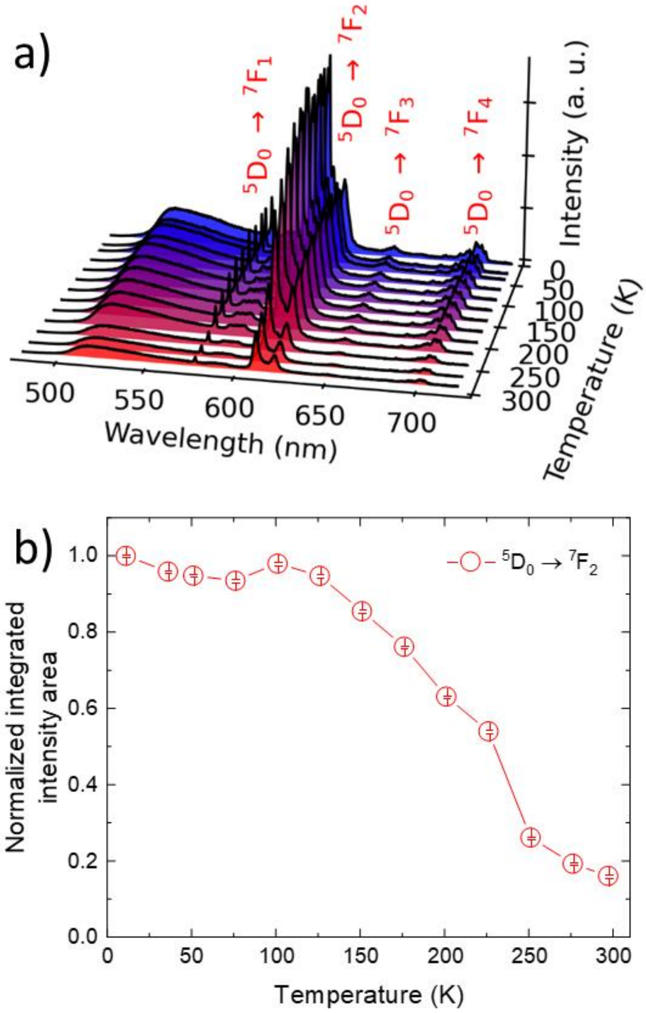

Figure S8. a) Emission spectra performed with the excitation at 464 nm in solid state in the temperature range 11 – 300 K for **2**. b) Normalized integrated intensity area of transition $^5D_0 \rightarrow {}^7F_2$ within the temperature range from 11 K to 300 K.

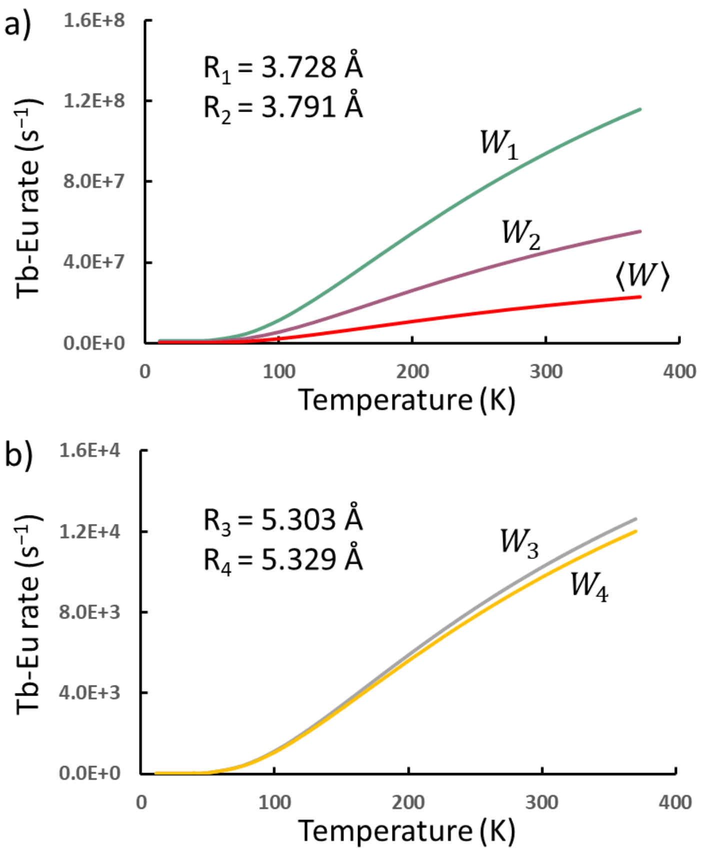

Figure S9. Energy transfer rates in the {Ln_4} cluster as a function of temperature. (a) shows the first two shortest distances, while (b) shows the longest two distances. The average energy transfer is shown in (A).

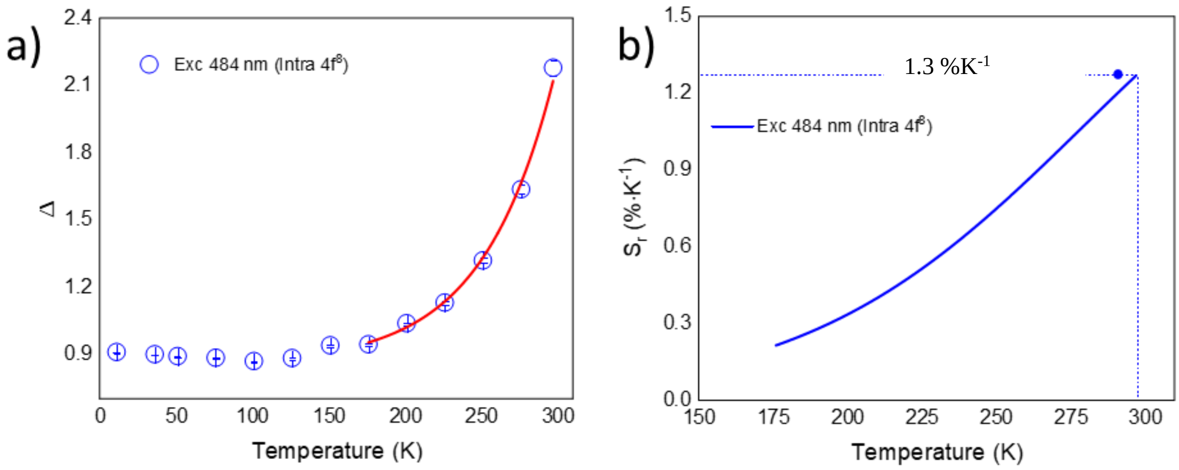

Figure S10. a) Temperature dependence of the normalized $^5D_4 \rightarrow {}^7F_5/{}^5D_0 \rightarrow {}^7F_2$ ratio performed for the emission spectra under the excitation at 484 nm in the temperature range 11 – 300 K for **2** with 3 experimental cycles (circles) and the associated fit (red curve) with a single exponential function (full line) (r>0.99); b) Temperature dependence of the corresponding thermal sensitivity (S_r).

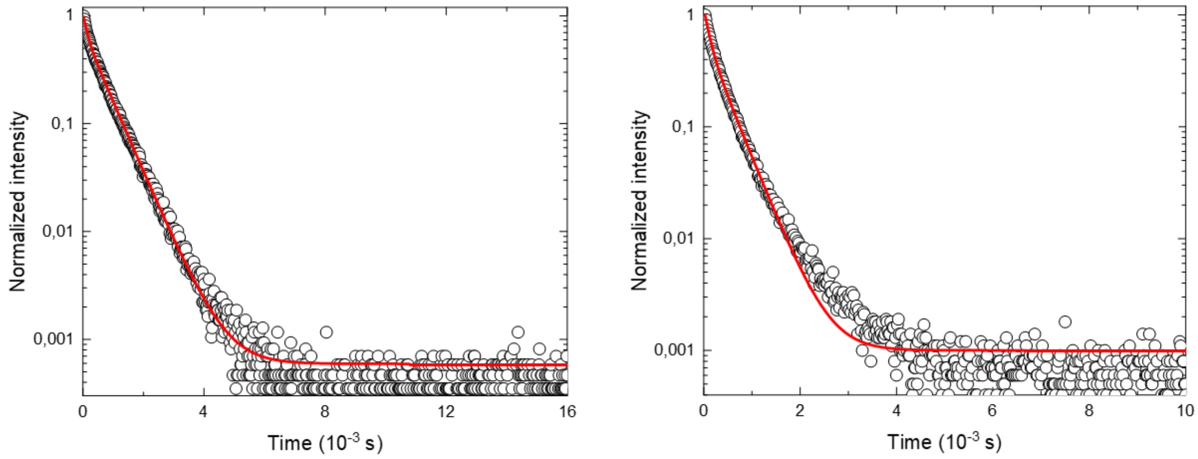

Figure S11. Temporal decay traces of 2 monitoring at (left) 488 nm ($^5D_4$) (a) and (right) 703 nm ($^5D_0$) (b) upon 330 nm excitation recorded at room temperature. The red solid curves represent double-exponential functions as the best fits to the experimental data (r>0.99).

3 Tables

Table S1. Crystal data and structure refinement for **1-3**.

Identification code	**1** • $2C_2H_3N$	**2** • $2C_2H_3N$	**3** • $2C_2H_3N$
Empirical formula	$C_{80}H_{92}N_4O_{26}Si_8Tb_4$	$C_{80}H_{92}N_4O_{26}Si_8EuTb_3$	$C_{80}H_{92}N_4O_{26}Si_8Gd_4$
Formula weight	2386.02	2379.06	2379.30
Temperature, K	100	100	100
Crystal size, mm	0.12 × 0.15 × 0.21	0.10 × 0.12 × 0.15	0.11 × 0.14 × 0.19
Crystal system	Monoclinic	Monoclinic	Monoclinic
λ, Å	0.71073	0.71073	1.54184
Space group	$P2_1/n$	$P2_1/n$	$P2_1/n$
a, Å	16.0660(3)	16.0131(3)	16.0244(2)
b, Å	15.3595(3)	15.3475(2)	15.3821(1)
c, Å	19.3080(4)	19.3288(4)	19.3780(2)
α, deg.	90	90	90
β, deg.	108.519(1)	108.276(2)	108.454(1)
γ, deg.	90	90	90
V, Å^3	4517.84(16)	4510.65(15)	4530.85(8)
Z	2	2	2
Density (calc.), g/cm^3	1.754	1.752	1.744
Absorption coefficient, mm^{-1}	3.275	3.192	20.288
F(000)	2360	2356	2352
Theta range, deg.	1.96 – 32.62	2.22 – 35.38	3.13 – 77.90
Index ranges	$-24 \le h \le 24$<br>$-23 \le k \le 23$<br>$-29 \le l \le 29$	$-25 \le h \le 24$<br>$-24 \le k \le 24$<br>$-30 \le l \le 29$	$-20 \le h \le 20$<br>$-18 \le k \le 19$<br>$-22 \le l \le 24$
Reflections collected	211519	90383	65689

Independent reflections, R_{int}	16472, 0.0275	18537, 0.0611	9580, 0.0700
Reflections with $I > 2\sigma(I)$	15782	14485	9138
Parameters refined	560	566	560
R_1 / wR_2 for $I > 2\sigma(I)$	0.0166 / 0.0410	0.0343 / 0.0653	0.0376 / 0.0970
R_1 / wR_2 for all data	0.0178 / 0.0415	0.0544 / 0.0697	0.0393 / 0.0983
Goodness-of-fit on F^2	1.054	1.004	1.030
T_{min}, T_{max}	0.532, 0.666	0.624, 0.717	0.003, 0.104
$\Delta\rho_{max}$ / $\Delta\rho_{min}$, e·Å^{-3}	1.361 / -0.688	1.525 / -1.015	1.430 / -1.933

Table S2. Main distances and angles for **1-3**.

Sample	Ln-O distance, Å	Ln-N distance, Å	Ln-Ln distance, Å	∠ O-Ln-O, °
**1**	2.3122(9)-2.3644(9),<br>2.4510(11) – EtOH,<br>2.2885(11)-2.3967(10) – acac	2.5537(13)	3.72723(10)-3.78998(11),<br>5.3025(1)-5.3288(1)	70.32(3)-73.86(3),<br>79.72(3)-81.66(3)
**2**	2.2757(17)-2.379(2) – Tb,<br>2.277(5)-2.442(4) – Eu,<br>2.453(2) – Tb1-EtOH,<br>2.409(6) – Eu1-EtOH,<br>2.305(2)-2.372(2) –<br>Tb-acac,<br>2.178(4)-2.439(5) –<br>Eu-acac	2.456(5) – Eu2,<br>2.569(2) – Tb2	3.6714(17)-3.838(6),<br>5.266(3)-5.268(3) – Tb…Tb,<br>5.307(7)-5.379(7) – Eu…Tb	69.50(17)-74.36(6),<br>78.34(13)-82.59(7)

**3**	2.309(2)–2.369(2),<br><br>2.452(3) – EtOH,<br><br>2.283(3)-2.396(2) – acac	2.558(3)	3.7320(3)-3.7905(3),<br><br>5.3073(4)-5.3315(4)	70.19(8)-73.69(8),<br><br>79.73(8)-81.75(8)

Table S3. A) Direct intramolecular energy transfer rates from the T_1 to the $^5D_4$ from the rising of the population in $^7F_5$ and $^7F_6$ levels: Δ is the donor–acceptor energy difference; $\boldsymbol{W_{d-d}}$, $\boldsymbol{W_{d-m}}$, and $\boldsymbol{W_{ex}}$ are the dipole-dipole, dipole-multipole, and exchange mechanisms (Eqs. S5 – S7) while $\boldsymbol{W}$ is the sum of these mechanisms in the same pathway (Eq. S9); $\boldsymbol{\phi}$ is the sum of these pathways, which is mainly constituted by the exchange mechanism of the $T_1 \rightarrow [^7F_5 \rightarrow {}^5D_4]$ pathway. B) Multiphonon decay rates from $^5D_3$ to $^5D_4$ considering N-phonons involved in the process (N from 2 to 5): Δ is the $^5D_3$–$^5D_4$ energy difference[19]; $\boldsymbol{\hbar\bar{\omega}}$ is the mean phonon energy; the $\boldsymbol{\alpha}$ factor was estimated from Miyakawa-Dexter approach [5] (Eq. S2); $\boldsymbol{W_{mp}}$ is the multiphonon rate between $^5D_3$–$^5D_4$ levels of Tb^{3+}.

A) Intramolecular Energy Transfer					
Pathway	Δ (cm^{-1})	W_{d-d} (s^{-1})	W_{d-m} (s^{-1})	W_{ex} (s^{-1})	W (s^{-1})
$T_1 \rightarrow [^7F_6 \rightarrow {}^5D_4]$	4872	2.500×10^{1}	2.500×10^{1}	0.000	2.500×10^{1}
$T_1 \rightarrow [^7F_5 \rightarrow {}^5D_4]$	6920	1.542×10^{-1}	1.631×10^{2}	1.895×10^{6}	1.896×10^{6}
					$\phi = 1.896\times10^{6}\ s^{-1}$
B) Multiphonon decay rates					
Pathway	Δ (cm^{-1})	N	$\hbar\bar{\omega}$ (s^{-1})	α (cm)	W_{mp} (s^{-1})
$^5D_3 \rightarrow {}^5D_4$	5792	2	2896	1.245×10^{-3}	7.389×10^{3}
$^5D_3 \rightarrow {}^5D_4$	5792	3	1930	2.077×10^{-3}	5.951×10^{1}
$^5D_3 \rightarrow {}^5D_4$	5792	4	1448	2.968×10^{-3}	0.341
$^5D_3 \rightarrow {}^5D_4$	5792	5	1158	3.903×10^{-3}	1.520×10^{-3}

Table S4. Pairwise Tb-to-Eu energy transfer rates for the shortest Tb-Eu distance (3.728 Å) at 300 K.

Pathway	Donor	Acceptor	Δ	W_{d-d}	W_{d-q}	W_{q-q}	W_{ex}	W_{md-md}	ω
1	$^5D_4 \rightarrow ^7F_6$	$^7F_1 \rightarrow ^5D_0$	3521	0	0	0	2.3E-22	0	7.4E-23
2	$^5D_4 \rightarrow ^7F_6$	$^7F_0 \rightarrow ^5D_0$	3149	2.9E-26	1.5E-22	3.5E-19	2.1E-16	0	3.6E-19
3	$^5D_4 \rightarrow ^7F_6$	$^7F_1 \rightarrow ^5D_1$	1787	1.2E-10	6.0E-07	1.4E-03	3.3E+00	0	2.2E+00
4	$^5D_4 \rightarrow ^7F_5$	$^7F_1 \rightarrow ^5D_0$	1473	0	0	0	9.6E+02	3.1E-04	3.2E+02
5	$^5D_4 \rightarrow ^7F_6$	$^7F_0 \rightarrow ^5D_1$	1415	0	0	0	2.4E+03	0	1.6E+03
6	$^5D_4 \rightarrow ^7F_5$	$^7F_0 \rightarrow ^5D_0$	1101	1.3E-04	7.7E-01	5.1E+03	1.9E+05	0	3.3E+02
7	$^5D_4 \rightarrow ^7F_4$	$^7F_1 \rightarrow ^5D_0$	206	0	0	0	1.3E+08	1.6E+01	4.2E+07
8	$^5D_4 \rightarrow ^7F_4$	$^7F_0 \rightarrow ^5D_0$	-166	2.3E-02	1.1E+02	5.1E+04	1.4E+08	0	1.0E+05
9	$^5D_4 \rightarrow ^7F_5$	$^7F_1 \rightarrow ^5D_1$	-10	2.9E-02	1.7E+02	1.1E+06	1.6E+08	4.2E-01	5.1E+07
10	$^5D_4 \rightarrow ^7F_5$	$^7F_0 \rightarrow ^5D_1$	-633	0	0	0	1.8E+07	3.9E+00	5.6E+05
11	$^5D_4 \rightarrow ^7F_6$	$^7F_1 \rightarrow ^5D_2$	-669	0	0	0	1.4E+07	0	1.8E+05
12	$^5D_4 \rightarrow ^7F_3$	$^7F_1 \rightarrow ^5D_0$	-773	0	0	0	5.9E+06	7.9E+00	4.8E+04
13	$^5D_4 \rightarrow ^7F_6$	$^7F_0 \rightarrow ^5D_2$	-1041	5.4E-05	2.8E-01	6.6E+02	4.0E+05	0	1.8E+03
14	$^5D_4 \rightarrow ^7F_3$	$^7F_0 \rightarrow ^5D_0$	-1145	1.5E-05	8.3E-02	4.6E+02	1.1E+05	0	7.8E-01
15	$^5D_4 \rightarrow ^7F_4$	$^7F_1 \rightarrow ^5D_1$	-1528	1.7E-08	8.0E-05	3.7E-02	3.9E+02	3.8E-07	8.4E-02
16	$^5D_4 \rightarrow ^7F_4$	$^7F_0 \rightarrow ^5D_1$	-1900	0	0	0	3.3E-01	2.8E-08	2.4E-05
17	$^5D_4 \rightarrow ^7F_3$	$^7F_1 \rightarrow ^5D_1$	-2507	4.0E-18	2.3E-14	1.2E-10	1.2E-07	1.3E-15	2.3E-13
18	$^5D_4 \rightarrow ^7F_5$	$^7F_1 \rightarrow ^5D_2$	-2717	0	0	0	2.7E-10	3.4E-18	2.0E-16
19	$^5D_4 \rightarrow ^7F_3$	$^7F_0 \rightarrow ^5D_1$	-2879	0	0	0	1.8E-12	1.7E-18	1.2E-18
20	$^5D_4 \rightarrow ^7F_5$	$^7F_0 \rightarrow ^5D_2$	-3089	1.2E-24	6.7E-21	4.4E-17	1.7E-15	0	4.3E-22
21	$^5D_4 \rightarrow ^7F_4$	$^7F_1 \rightarrow ^5D_2$	-3984	0	0	0	9.6E-31	4.6E-39	1.6E-39
22	$^5D_4 \rightarrow ^7F_4$	$^7F_0 \rightarrow ^5D_2$	-4356	5.4E-48	2.6E-44	1.2E-41	3.3E-38	0	1.8E-47
23	$^5D_4 \rightarrow ^7F_3$	$^7F_1 \rightarrow ^5D_2$	-4963	0	0	0	7.8E-52	4.1E-59	1.2E-62
24	$^5D_4 \rightarrow ^7F_3$	$^7F_0 \rightarrow ^5D_2$	-5335	6.1E-71	3.4E-67	1.9E-63	4.6E-61	0	2.4E-72
								$\sum \omega =$	**9.4E+07**

Table S5. Simulated Tb-Eu pair occurrence ($\boldsymbol{O_i}$), pairwise energy transfer ($\boldsymbol{W_i}$), and Tb-Eu average energy transfer ($\langle \boldsymbol{W} \rangle_i$) for the 4th first Tb-Eu distances.

$\boldsymbol{i}$	**Tb-Eu distance (Å)**	$\boldsymbol{O_i}$	$\boldsymbol{W_i}$ **(s^{-1})**	$\langle \boldsymbol{W} \rangle_i$ **(s^{-1})**
1	3.728	0.714 ± 0.004	9.43×10^7	$1.3 \pm 0.1 \times 10^7$
2	3.791	0.731 ± 0.004	4.49×10^7	$6.2 \pm 0.7 \times 10^6$
3	5.303	0.348 ± 0.003	1.02×10^4	$7 \pm 1 \times 10^2$
4	5.329	0.348 ± 0.003	9.75×10^3	$6 \pm 1 \times 10^2$

4 References

1 R. Reisfeld and C. K. Jørgensen, *Lasers and Excited States of Rare Earths*, Springer-Verlag Berlin Heidelberg, 1st edn., 1977, vol. I.

2 H. W. Moos, *J. Lumin.*, 1970, **1**–**2**, 106–121.

3 B. Z. Malkin, in *Spectroscopic Properties of Rare Earths in Optical Materials*, eds. R. Hull, J. Parisi, R. M. Osgood, H. Warlimont, G. Liu and B. Jacquier, Springer Berlin Heidelberg, Berlin, Heidelberg, 2005, pp. 130–190.

4 L. A. Riseberg and M. J. Weber, in *Progress in Optics*, 1977, vol. 14, pp. 89–159.

5 T. Miyakawa and D. L. Dexter, *Phys. Rev. B*, 1970, **1**, 2961–2969.

6 N. Yamada, S. Shionoya and T. Kushida, *J. Phys. Soc. Japan*, 1972, **32**, 1577–1586.

7 W. H. Fonger and C. W. Struck, *J. Lumin.*, 1978, **17**, 241–261.

8 F. Auzel, G. F. De Sá and W. M. de Azevedo, *J. Lumin.*, 1980, **21**, 187–192.

9 A. De, M. A. Hernández-Rodríguez, A. N. Carneiro Neto, V. Dwij, V. Sathe, L. D. Carlos and R. Ranjan, *J. Mater. Chem. C*, 2023, **11**, 6095–6106.

10 A. N. Carneiro Neto, E. Kasprzycka, A. S. Souza, P. Gawryszewska, M. Suta, L. D. Carlos and O. L. Malta, *J. Lumin.*, 2022, **248**, 118933.

11 O. L. Malta, *J. Lumin.*, 1997, **71**, 229–236.

12 O. L. Malta and F. R. Gonçalves e Silva, *Spectrochim. Acta Part A Mol. Biomol. Spectrosc.*, 1998, **54**, 1593–1599.

13 R. Longo, F. R. Gonçalves e Silva and O. L. Malta, *Chem. Phys. Lett.*, 2000, **328**, 67–74.

14 O. L. Malta, *J. Non. Cryst. Solids*, 2008, **354**, 4770–4776.

15 A. N. Carneiro Neto, E. E. S. Teotonio, G. F. de Sá, H. F. Brito, J. Legendziewicz, L. D. Carlos, M. C. F. C. Felinto, P. Gawryszewska, R. T. Moura Jr., R. L. Longo, W. M. Faustino and O. L. Malta, in *Handbook on the Physics and Chemistry of Rare Earths, volume 56*, eds. J.-C. G. Bünzli and V. K. Pecharsky, Elsevier, 2019, pp. 55–162.

16 O. L. Malta, *Chem. Phys. Lett.*, 1982, **88**, 353–356.

17 O. L. Malta, *Chem. Phys. Lett.*, 1982, **87**, 27–29.

18 R. T. Moura Jr., A. N. Carneiro Neto, E. C. Aguiar, C. V. Santos-Jr., E. M. de Lima, W. M. Faustino, E. E. S. Teotonio, H. F. Brito, M. C. F. C. Felinto, R. A. S. Ferreira, L. D. Carlos, R. L. Longo and O. L. Malta, *Opt. Mater. X*, 2021, **11**, 100080.

19 W. T. Carnall, H. Crosswhite and H. M. Crosswhite, *Energy level structure and transition probabilities in the spectra of the trivalent lanthanides in LaF_3,* Argonne, IL, United States, 1978.

20 S. Edvardsson and M. Klintenberg, *J. Alloys Compd.*, 1998, **275**–**277**, 230–233.

21 A. N. Carneiro Neto and R. T. Moura Jr., *Chem. Phys. Lett.*, 2020, **757**, 137884.

22 F. R. G. e Silva and O. L. Malta, *J. Alloys Compd.*, 1997, **250**, 427–430.

23 G. S. Ofelt, *J. Chem. Phys.*, 1963, **38**, 2171–2180.

24 E. Kasprzycka, A. N. Carneiro Neto, V. A. Trush, L. Jerzykiewicz, V. M. Amirkhanov, O. L. Malta, J. Legendziewicz and P. Gawryszewska, *J. Rare Earths*, 2020, **38**, 552–563.

25 T. Kushida, *J. Phys. Soc. Japan*, 1973, **34**, 1318–1326.

26 A. N. Carneiro Neto, R. T. Moura, A. Shyichuk, V. Paterlini, F. Piccinelli, M. Bettinelli and O. L. Malta, *J. Phys. Chem. C*, 2020, **10**, 10105–10116.

27 V. Trannoy, A. N. Carneiro Neto, C. D. S. Brites, L. D. Carlos and H. Serier-Brault, *Adv. Opt. Mater*., 2021, **9**, 2001938.

28 D. L. Dexter, *J. Chem. Phys.*, 1953, **21**, 836–850.

29 I. M. D. Galanin and I. M. Frank, *Zh. Eksperim. i Teor. Fiz*, 1951, **21**, 114–120.

30 V. L. Ermolaev and E. B. Sveshnikova, *J. Lumin.*, 1979, **20**, 387–395.

31 E. B. Sveshnikova and V. L. Ermolaev, *Opt. Spectrosc.*, 2011, **111**, 34–50.

32 P. A. Tanner, M. Chua and M. F. Reid, *J. Alloys Compd.*, 1995, **225**, 20–23.

33 M. Chua, P. A. Tanner and M. F. Reid, *J. Lumin.*, 1994, **58**, 356–360.

34 M. Chua, P. A. Tanner and M. F. Reid, *Solid State Commun.*, 1994, **90**, 581–583.

35 G. S. Ofelt, *J. Chem. Phys.*, 1963, **38**, 2171–2180.

36 O. L. Malta, H. F. Brito, J. F. S. Menezes, F. R. G. e Silva, S. Alves, F. S. Farias and A. V. M. de Andrade, *J. Lumin.*, 1997, **75**, 255–268.

37 O. L. Malta, H. J. Batista and L. D. Carlos, *Chem. Phys.*, 2002, **282**, 21–30.

38 R. T. Moura Jr., A. N. Carneiro Neto, R. L. Longo and O. L. Malta, *J. Lumin.*, 2016, **170**, 420–430.

39 A. S. Souza, L. A. O. Nunes, I. G. N. Silva, F. A. M. Oliveira, L. L. Da Luz, H. F. Brito, M. C. F. C. Felinto, R. A. S. Ferreira, S. A. Júnior, L. D. Carlos and O. L. Malta, *Nanoscale*, 2016, **8**,

5327–5333.

40 M. T. Berry, P. S. May and Q. Hu, *J. Lumin.*, 1997, **71**, 269–283.
